# Supplementary material for: Transition program for adolescents with congenital heart disease in transition to adulthood: protocol for a mixed-method process evaluation study (the STEPSTONES project)
Source: BMJ Open. 2019 Aug 2;9(8):e028229. doi: 10.1136/bmjopen-2018-028229 (PMC6687006; doi:10.1136/bmjopen-2018-028229)
Supplement: Author's manuscript [file bmjopen-2018-028229.draft_revisions.pdf]

BMJ Open is committed to open peer review. As part of this commitment we make the peer review history of every article we publish publicly available.

When an article is published we post the peer reviewers' comments and the authors' responses online. We also post the versions of the paper that were used during peer review. These are the versions that the peer review comments apply to.

The versions of the paper that follow are the versions that were submitted during the peer review process. They are not the versions of record or the final published versions. They should not be cited or distributed as the published version of this manuscript.

BMJ Open is an open access journal and the full, final, typeset and author-corrected version of record of the manuscript is available on our site with no access controls, subscription charges or pay-per-view fees (http://bmjopen.bmj.com).

If you have any questions on BMJ Open's open peer review process please email info.bmjopen@bmj.com

BMJ Open

Transition program for adolescents with congenital heart disease in transition to adulthood: protocol for a mixed-methods process evaluation study (the STEPSTONES project)

Journal:	*BMJ Open*
Manuscript ID	bmjopen-2018-028229
Article Type:	Protocol
Date Submitted by the Author:	28-Nov-2018
Complete List of Authors:	Saarijärvi, Markus; Goteborgs Universitet, Institute of Health and Care Sciences; KU Leuven, Department of Public Health and Primary Care<br>Wallin, Lars; Hogskolan Dalarna, School of Education, Health and Social studies; Goteborgs Universitet, Institute of Health and Care Sciences<br>Moons, Philip; KU Leuven, Department of Public Health and Primary Care; Goteborgs Universitet, Institute of Health and Care Sciences<br>Gyllensten, Hanna ; Goteborgs Universitet, Institute of Health and Care Sciences; Goteborgs Universitet, University of Gothenburg Centre for Person-Centered Care (GPCC)<br>Bratt, Ewa-Lena; Goteborgs Universitet, Institute of Health and Care Sciences; Drottning Silvias barn- och ungdomssjukhus i Goteborg, Department of Pediatric Cardiology
Keywords:	Congenital heart disease < CARDIOLOGY, Adolescents, Process evaluation, Randomized controlled trial, Implementation, Transition

SCHOLARONE™
Manuscripts

Transition program for adolescents with congenital heart disease in transition to adulthood: protocol for a mixed-methods process evaluation study (the STEPSTONES project)

Markus Saarijärvi MSc, RN[1, 2], Lars Wallin PhD, RN[1, 3-4], Philip Moons PhD, RN[1-2, 5], Hanna Gyllensten PhD, RPH[1, 6], Ewa-Lena Bratt PhD, RN[1, 7]

[1] Institute of Health and Care Sciences, University of Gothenburg, Gothenburg, Sweden

[2] KU Leuven, Department of Public Health and Primary Care, Leuven, Belgium

[3] School of Education, Health and Social Studies, Dalarna University, Falun, Sweden

[4] Department of Neurobiology, Care Sciences and Society, Karolinska Institute, Stockholm, Sweden

[5] Department of Paediatrics and Child Health, University of Cape Town, Cape Town, South Africa

[6] University of Gothenburg Centre for Person-Centered Care (GPCC), Sahlgrenska Academy, University of Gothenburg, Gothenburg, Sweden

[7] Department of Pediatric Cardiology, The Queen Silvia Children's Hospital, Gothenburg, Sweden

Markus Saarijärvi: markus.saarijarvi@gu.se

Lars Wallin: lars.wallin@du.se

Philip Moons: philip.moons@kuleuven.be

Hanna Gyllensten: hanna.gyllensten@gu.se

Ewa-Lena Bratt: ewa-lena.bratt@gu.se

Corresponding author:

Markus Saarijärvi, MSc, RN, Institute of Health and Care Sciences, University of Gothenburg, PO-Box 457, 405 30 Gothenburg, Sweden; Tel: +46 766 61 58, markus.saarijarvi@gu.se

Word count: 3760

For peer review only

ABSTRACT

Introduction

Today, the majority of young persons living with chronic conditions in high-income countries survive into adulthood and will need life-long medical follow-up. Therefore, transition programs have been developed to facilitate transfer to adult care, and to support self-management and independence during adulthood. The STEPSTONES project aims to evaluate the effectiveness of a person-centered transition program for empowering adolescents with congenital heart disease in transition to adulthood. To understand how the transition program causes change and how outcomes are created, process evaluation is imperative to assess implementation, context and mechanisms of impact. This protocol aims to describe the process evaluation of the STEPSTONES transition program.

Methods and design

Medical Research Council guidance for Process evaluation of complex interventions will be the guiding framework for this mixed-methods study. The combination of qualitative and quantitative data will capture different aspects of program delivery. The sample will consist of participants in the STEPSTONES randomized controlled trial (RCT), persons implementing the program, and healthcare professionals. Quantitative data will consist of protocols and routine monitoring documents from the RCT, data collected from patient registries and socio-demographic data to assess the implementation of the intervention. This data will be analyzed with quantitative content analysis, along with descriptive and inferential statistics. Qualitative data will consist of participatory observations, logbooks, and interviews with persons implementing the program, participants and healthcare professionals. Analyses will be performed using qualitative content analysis to investigate mechanism of impact, context and delivery. Quantitative and qualitative data will be integrated in the final stage by using a triangulation protocol according to mixed-methods guidelines.

Ethics and dissemination

The study is approved by the Regional Ethical Review Board in Gothenburg, Sweden. Results will be presented in open-access, peer-reviewed journals and at international scientific conferences.

Keywords

For peer review only

Adolescents, congenital heart disease, process evaluation, randomized controlled trial, implementation, transition.

ARTICLE SUMMARY

Strengths and limitations of this study

- This is the first process evaluation study of a transition program for adolescents with congenital heart disease. Therefore, the study is expected to fill a gap in current knowledge on how transition programs produce change.
- The use of multiple data sources, both quantitative and qualitative, strengthens the quality of the findings by triangulation between data sources.
- Quantitative data in this study rely heavily on routine monitoring documents from the randomized controlled trial, which may affect the trustworthiness of the process evaluation results.
- Process evaluation data will be reported separately from the results from the effectiveness evaluation.

For peer review only

INTRODUCTION

Today, the majority of young persons living with chronic conditions (CC) in high-income countries survive into adulthood and need life-long follow up.[1, 2] When going from adolescence to adulthood, these young people will face challenges beyond those normally associated with this period in life.[3, 4] Thus, transition programs have been advocated by several guidelines and consensus statements to ensure continuity of care and support self-management and independence in adulthood.[5-7] The current knowledge emphasizes that such programs should be individually tailored, developmentally appropriate and encompass both the physical and psychosocial needs of the adolescent.[8-9] Furthermore, they should include strategies facilitating both transition as a developmental process, and the transfer of care from a pediatric to an adult context.[5] Transition programs have been evaluated to some extent during the last decade, showing improvements in outcomes such as patients' knowledge about their condition, increased self-management, reduced delay in accessing adult care and improved disease-specific indicators.[10-13] However, only four studies evaluating transition programs were included in the recent review by the Cochrane Database of Systematic Reviews, and due to the studies lack of sufficient follow-up and rigorous design, the review concluded that existing research on transition programs is insufficient in proving hard evidence on effects. Moreover, none of these studies provided knowledge on the pathway of causal mechanisms leading to the desired outcomes.[13] More evidence is therefore pivotal in explaining causality and replicability of these interventions.

Transition programs are complex interventions, comprising numerous interacting ingredients. When the effectiveness of a complex intervention is evaluated, the active ingredients producing the outcome are unknown. Process evaluation is therefore indispensable in evaluating complex interventions, explaining the mechanism of impact and causal pathways.[14-16] Core elements of process evaluation studies are to investigate how complex interventions (e.g. transition programs) create change, how changes are affected by fidelity and potential moderators on the implementation process, and the impact of contextual factors and participants' engagement. Thus, process evaluation is not solely interested in whether the intended intervention produces effect, but also in unpacking "the black box" of the intervention to investigate how effects are made.[17] In addition to investigating aforementioned aspects, process evaluation studies should also explore if the intervention is evaluated properly with regards to internal and external validity of the randomized controlled trial (RCT), and if the intervention can be sustained in practice.[18] Therefore, the STEPSTONES (Swedish Transition Effects Project Supporting

For peer review only - http://bmjopen.bmj.com/site/about/guidelines.xhtml

Teenagers with chrONic mEdical conditionS) project was established with the purpose of developing, testing and evaluating a person-centered transition program for adolescents with congenital heart disease (CHD). This project will provide new evidence on the effects, causality and replicability of transition programs for adolescents with CCs.

The transition program

The STEPSTONES project is a person-centered transition program for adolescents (age 16-18.5 years) with CHD. The program was developed from a brief transition intervention previously tested in Belgium.[19] To adjust this intervention to the Swedish context, qualitative and quantitative preparatory studies were performed on the target population.[20-23] Furthermore, stakeholder involvement, literature review and behavior change theories were applied, following the protocol for developing health promoting programs, intervention mapping.[24] The program aims to be generic, and thus applicable to other CCs. However, CHD was chosen as the target population of this intervention, being the most common congenital malformation in need of life-long follow up.[25] The program consists of eight key components, implemented in five steps (see Fig. 1). A transition coordinator (TC) operating at each intervention center delivers the program. The TC is a specialized, pediatric nurse who has received tailored training in delivering the intervention.

Participants and setting – RCT-study

The STEPSTONES program is evaluated through a hybrid RCT, where a longitudinal observational study is embedded within the RCT (Fig. 2). The study is performed in seven University hospitals in Sweden where two centers consist of an intervention group (n=70), and a comparison group (n=70) where individual randomization is performed to either of the groups. The remaining five centers constitute additional control groups (n=70) where no contamination of the intervention is expected. The hypothesis is that adolescents randomized to receive the transition program over a two-year period will have a higher patient empowerment score compared to those randomized to usual care. Inclusion criteria for participants in the RCT-study are: 16 years of age with CHD, Swedish speaking and literate. Their parents will also be invited to participate. A study protocol describing the RCT has been published.[26]

Outcome measures of the intervention – RCT-study

The primary outcome of patient empowerment is measured using the GYPES (Gothenburg Young Persons Empowerment Scale).[27] Patient empowerment is defined as "an enabling

For peer review only

process or outcome arising from communication with the healthcare professional and a mutual sharing of resources over information relating to illness, which enhances the patient's feelings of control, self-efficacy, coping abilities and ability to achieve change over their condition".[28] The secondary outcomes are transition readiness, disease-related knowledge, health behaviors, patient-reported health, quality of life, healthcare usage and parental uncertainty towards transfer to adult care.[26]

Process evaluation of the transition program

In accordance with the Medical Research Council's (MRC) guidance on process evaluation of complex interventions, key aspects of this evaluation are centered on the logic model of the intervention, the implementation, mechanism of impact and context.[17] These aspects have a direct impact on how effectively interventions work in producing the desired outcome. The logic model of the intervention provides a structure for designing, carrying out and analyzing process data.[17, 29] This model can also work as a blueprint of the intervention, describing the underlying theoretical assumptions, input (e.g. theory, training and resources), how the intervention is intended to be delivered, intervention components and how the intervention aims to cause change.[17] In Fig. 3, the logic model of the program (grey box) is presented along with the key concepts in process evaluation according to the MRC guidance.[17] This figure provides an overview, starting from the logic model, on how the process evaluation of the STEPSTONES transition program is structured.

The concept of implementation within this model focuses on how successful delivery of the intervention is accomplished and what is actually being delivered (i.e. fidelity). Fidelity is described as "the degree to which the clinical intervention was delivered as it was intended" and is assessed by evaluating adherence, dose and reach of each component of the intervention.[30] Fidelity may have a direct impact on outcomes, and thus researchers cannot determine if an intervention failed due to poor implementation or to inadequacies in the program, unless fidelity is measured.[31]

The concept of mechanism of impact refers to how the intervention activities produce intended or unintended effects,[17] in this case the main outcome of patient empowerment. This can be studied by including data on participants' responsiveness and interaction with the intervention, their trajectory of care within the intervention, and by assessing possible moderators such as quality of delivery and complexity of the intervention.[17, 30]

For peer review only

Finally, context includes factors external to the intervention, which influences implementation, or whether the mechanisms of impact act as intended within the context in which the intervention is being delivered.[17] Contextual factors have shown to impact how the intervention is adapted locally and in addition, shape the outcomes of the intervention.[32]

By exploring the relationship between the logic model, how the intervention is delivered in practice and relating this to the process evaluation concepts, the outcomes of the intervention can be understood.[30] Therefore, the overall aim is to perform a process evaluation study, assessing the implementation of the program and the contextual impact on this process in order to understand and explain the mechanisms creating the outcome of the transition program. Research questions for the process evaluation of the transition program are listed together with proposed research methods in Fig. 4.

METHOD

A mixed-methods embedded design will be used, including collection, analysis and integration of quantitative and qualitative data.[33] During the designing and planning of the data collection the process evaluation guidance by the MRC[14] was chosen as the methodological framework.

Participants and setting

For the quantitative assessment, participants (n=70) and participating parents in the intervention group will be included. In addition, the TCs at the intervention centers, as well as a sample of physicians and nurses at all participating centers will be asked to participate. For the qualitative assessment, we will use a purposive sub-sample according to qualitative methodological guidelines. Specifically, we will strive for variability of the participants' characteristics in order to capture a breadth of experiences.[34]

Data collection

Data will consist of a range of sources in order to capture different perspectives of key process variables. Quantitative and qualitative data collection will be carried out simultaneously and synthesized in the final analysis.

Quantitative data collection

The following data sources will be used to collect quantitative data on process measures:

For peer review only

1) *Enrolment form*
   - The enrolment form is based on the five delivery steps of the intervention (see Fig. 1). The data allows for measurement of adherence and attendance in the intervention for all eligible participants. Drop out from the study, non-attendance for visits and reasons for this will also be registered, which gives information about acceptability of the transition program.
2) *Intervention implementation form*
   - This form will be filled out by the TC after each individual patient encounter, whether in person, by telephone, text message or email. This form contains the components of the intervention, e.g. information and education about the health condition, treatment and health behavior, and information about the adult care follow-up. It also gives information about topics covered, supportive activities and recommendations given, providing an indication about adherence, and dose delivered of the intervention to the patients. The data source will also give information on which communication channel is primarily used for contact between the TC and patients.
3) *Adverse event report (AE)*
   - The occurrence of AE's will give an indication of potential attrition from the study.
4) *Transition plans*
   - Each patient in the intervention receives a transition plan in which the care is documented. An analysis of this plan will be undertaken to understand what the main objectives were in the transition of individual patients. This analysis will estimate which components of the intervention have been delivered, the outcomes of these, and how this is documented. The data from these sources will give information about possible mechanism of impact, adherence, dose and the potential effects of moderating factors on fidelity such as patient characteristics.
5) *Socio-demographic data*
   - Age, sex, education level, diagnosis and pharmacotherapy, comorbidities, geographic location, living situation, country of birth, parental marital status and employment situation will be retrieved from the Swedish registry of congenital heart disease (Swedcon) and the questionnaires used in the RCT. This data will be used to examine the characteristics of participants who engage with and

For peer review only

continue participation in the intervention, and those who do not (e.g. drop-outs and non-participants), and how this affects the reach of the program.

6) *Time-management form*
   - In this form, the TCs register how much time is spent on performing different tasks of the transition program, such as preparation of visits, patient contacts, administration and contacts with other healthcare professionals. Time spent on study-related tasks (e.g. recruitment, randomization and data-entry) is excluded from this form. By collecting data on time-management, differences in intervention delivery between the two centers will be visible, also giving valuable information to a future implementation scenario.

Qualitative data collection

The qualitative data will be used to answer questions on how implementation is achieved, possible moderating factors and how the participants experience and engage with the intervention. Qualitative data will be collected and analyzed by a researcher (MS) that is not involved in the RCT-study, to ensure trustworthiness of the data.[17] The following qualitative data sources will be used:

1) *Pair-interviews with TC*
   - Throughout the course of the trial, pair-interviews[36] with the TCs will be performed every six months. An interview guide will be used covering topics such as the experiences of implementing the intervention, barriers and facilitators and local contextual factors.
2) *Diary notes written by the TC*
   - Reflective diary notes are requested from the TCs, describing their experiences of implementing the transition program, barriers and facilitators and contextual factors they perceive to have affected the intervention.[37]
3) *Participatory observations*
   - Observations of outpatient visits will be performed to investigate how the transition program is being implemented in practice and the responsiveness and engagement of the participants. The observations will provide data on several process variables such as fidelity, adaptations, dose and participants engagement. Both a structured observation protocol and field notes will be used. The protocol includes components from the intervention implementation form and in addition, practical applications of how the intervention is intended to be

For peer review only

delivered, e.g. use of materials and methods to deliver the behavior change techniques and application of person-centered-care. Informal interviews will be conducted with the TCs and participants in connection with the observations as a way of ensuring trustworthiness.[38]

- Observations of an information day for adolescents and their parents, will be held between the second and third outpatient visit in the transition program. During the information day, adolescents and their parents will get the opportunity to meet their peers, receive information about the adult care and discuss issues regarding transfer and transition. Participatory observations of these meetings will be performed, including informal interviews with the participants about their experiences of the day.

4) *Semi-structured interviews with adolescents and parents*
   - Participants and their parents will be interviewed about their experiences one month after completion of the transition program. The interviews will give increased understanding of the dose received by the participants, potential moderating factors on intervention delivery, acceptability of the intervention and potential improvements for a future implementation of the transition program into current practice.

5) *Focus group interviews with the healthcare team*
   - Focus group interviews will be held with interdisciplinary teams from the intervention centers. The aim is to explore what contextual factors on a local and organizational level are perceived to affect implementation and what prerequisites that are needed for a future implementation of the transition program. The sample will consist of physicians and nurses from both pediatric cardiology and adult care with experiences of young persons with CHD.[39]

6) *Usual care assessment*
   - Throughout the course of the trial, assessment of the usual care in the control and comparison groups will be undertaken through participatory observations of outpatient visits and interviews with healthcare professionals. The objective is to assess current practices, barriers and facilitators for future implementation and identify possible contamination between study sites.

Data analysis

Quantitative data analysis

For peer review only

The process variables (e.g. adherence and dose) retrieved from the enrolment form, intervention implementation form and socio-demographic data will be entered into a database and analyzed in the Statistical Package for the Social Sciences (SPSS) by using descriptive statistics with frequency distributions, proportions, variability over time and means. The effect on process variables of intervention centers' characteristics and patients' socio-demographic factors will be analyzed by regression analysis, as will data from the time-management form, to explain potential moderators on fidelity. The reach of the intervention (i.e. did the intended population participate in the study), will be assessed by comparing participants versus non-participants of the RCT with socio-demographic data and clinical data (e.g. disease characteristics). The MRC guidance recommends reporting results of the process evaluation separately from the effectiveness evaluation to avoid biased interpretation of effectiveness results.[17] We are following this recommendation but will in addition perform a post-hoc regression analysis on dose-outcome relationships as well as sub-group analysis for relevant clinical and demographical variables.

The data from the transition plans will be analyzed using descriptive statistics along with quantitative content analysis[35] using the software NVivo (QSR International Pty Ltd. Version 12, 2018) by counting word occurrences related to the components of the intervention to summarize what the main objectives were of each individual participant's transition. Adverse events will be analyzed if they occur.

Qualitative data analysis

Design, sampling, analysis and reporting of the qualitative findings will follow the Consolidated Criteria for Reporting Qualitative research.[34] Qualitative data will be analyzed with manifest content analysis, as described by Graneheim and Lundman.[40] All qualitative data analysis will be performed in the software NVivo.

Integrating quantitative and qualitative data

Analysis and reporting of the mixed-method study will follow the Good Reporting of A Mixed Methods Study framework.[41] The mix of quantitative and qualitative data can give information about contextual factors that affect implementation and potential moderating factors on fidelity.[17] Qualitative data can be used to explain quantitative findings.[33] The integration of data will be performed by creating a triangulation protocol, which works as a scheme where the different data sources are combined and findings from different components of the process

For peer review only

evaluation are listed and compared. By looking for agreement and disagreement between the data sources the findings may lead to a better understanding of the results.[42]

Patient and public involvement

The STEPSTONES project was developed with the support of an advisory board and panel of international experts consisting of stakeholders, i.e. young adults with CHD, parents, clinicians, researchers and representatives from the patient organization. Patients will not be involved in the recruitment of this study; however, young adults with CHD will be involved during the adolescent day sharing their experiences of the transfer to adult care and transition to adulthood, acting as role models and providing peer support. Dissemination of results to study participants will be delivered through the patient organization and social media.

Ethics and dissemination

Adolescents, parents and health care professionals in the RCT have consented to participation after receiving written and oral information about the study. Written consent to participate in the qualitative data collection will be requested after additional written and oral information about the study. Ethical approval for the study was received from the Regional Ethical Review Board in Gothenburg, Sweden (No.931-15), and the study will be performed in accordance to the Declaration of Helsinki.[43] Findings of the process evaluation study will be published in open-access, peer-reviewed scientific journals and presented at national and international conferences.

DISCUSSION

Process evaluation studies have been increasing in recent years and are imperative to explaining how complex interventions produce the desired outcomes. Furthermore, such evaluation studies are arguably more important when interventions fail to produce the intended outcomes. Since its release in 2015, MRC guidance has been used in process evaluation studies, providing extensive data on important aspects of interventions delivery.[44-45] However, MRC does lack certain potentially important aspects, for example, the impact of recruitment and the internal and external validity of the RCT on outcomes. For this reason, we have added these research questions to our protocol.

For peer review only

The proposed study has several strengths. Firstly, when implementing a complex intervention, a clear description of the logic model of the intervention is beneficial, not only as a benchmark for evaluators but also for those delivering the intervention. Previous studies show that complex interventions with clear descriptions have a greater chance of being implemented with high adherence.[46] The logic model of our intervention was developed using the framework of intervention mapping, which provides a thorough evidence base.[24] In addition, the materials, resources and training for the TCs can facilitate strategies, enhancing intervention fidelity.[30] This process evaluation study can thus add to the existing evidence on how frameworks, such as intervention mapping, can aid in intervention development, implementation and evaluation.[24]

Secondly, the planned extent of data sources, methods and scope of data collection have, to the best of our knowledge, not been applied in previous process evaluation studies. This is beneficial for many reasons. The use of various sources can strengthen understanding of how different factors interact in creating outcomes. A recent review concluded that only one quarter of all studies measuring fidelity and engagement in interventions used multiple data collection methods such as observations and self-reported measures.[47] A range of qualitative methods within intervention studies can therefore strengthen the evaluation since these methods are forceful in capturing contextual factors.[32, 48] Moreover, quantitative data provides measures for important process variables such as fidelity.[30] By collecting data throughout the trial, this study can also handle the impact of 'teething problems' (e.g. TCs learning curves) by following changes over time and combining quantitative measures with stakeholder interviews.[17]

Thirdly, the findings of the process evaluation will be reported separately from the effectiveness evaluation of the STEPSTONES study, avoiding biased interpretations of results and therefore strengthening the validity of the findings.[17] Since the process measures of the study rely on routine monitoring data, validity of these documents has to be ensured by quality control and cross-checking between different sources. Routine monitoring data are effective in reducing response bias; they are cost-effective and allow for analysis over time.[17]

However, some challenges and limitations of this process evaluation study have to be considered. Factors affecting the recruitment are not directly included in the qualitative data collection. In Steckler and Linnan's[49] framework, this is a part of the process evaluation which is not included in the MRC guidance.[17] Recruitment pace, selection of study participants and recruiters' skills could arguably be considered moderating factors on reach and outcomes. That said, the acceptability of the intervention is explored and measured, and so is reach in terms of

For peer review only

participants approached and recruited. Furthermore, the TCs performing the intervention, and their performance during the intervention, will have a great impact on many of the key process measures since they are the only personnel delivering the intervention. To manage this, data will also be collected from participants and healthcare professionals in order to broaden the perspective. Process evaluators should also be aware of how they impact the quality of the collected data (e.g. Hawthorne-effects), especially in participatory observations.[50] However, a small sub-sample of qualitative observations from a total of 210 outpatient visits should arguably give little or no impact on effectiveness outcomes of the RCT. Nevertheless, it may affect the quality of the process evaluation results. To manage this, establishing good relations between the researchers performing the process evaluation and the TCs and healthcare professionals is important to get access to the field and retrieve trustworthy data. Informal interviews in connection with observations can also strengthen trustworthiness.[48]

Conclusions

Transfer of care for adolescents living with chronic conditions is a timely issue, and the effectiveness and causal assumptions of transition programs are yet to be proven. It is therefore imperative to conduct high-quality process evaluation studies to understand how the programs are delivered, for whom they are delivered and under what circumstances. The effect of contextual factors and possible moderating factors on intervention fidelity and participant engagement may also increase transferability of findings and can thus inform implementation of transition programs in other settings.

List of abbreviations

AE: Adverse Event

CHD: Congenital Heart Disease

MRC: Medical Research Council

RCT: Randomized Controlled Trial

STEPSTONES: Swedish Transition Effects Project Supporting Teenagers with chronic mEdical conditionS

SWEDCON: Swedish congenital heart disease registry

For peer review only

TC: Transition coordinator

Competing interests' statement

The authors declare that they have no competing interest.

Funding statement

This work was supported by research grants from the Swedish Heart-Lung Foundation (grant 20150535); Swedish Research Council for Health, Working Life and Welfare-FORTE (grant STYA-2015/0003); Swedish Children Heart Association; Swedish Research Council (grant 2015-02503); and the Institute of Health and Care Sciences of the University of Gothenburg.

Author contributions

MS, LW, PM, HG and ELB conceived the study and were responsible for the design and draft of the study protocol. All authors critically revised and approved the manuscript.

Author information

MS is a PhD student at the Institute of Health and Care Sciences, University of Gothenburg, Sweden and at the Academic Center for Nursing and Midwifery, Department of Public Health and Primary Care, KU Leuven, Belgium. LW is a Professor of Nursing Science at the School of Education, Health and Social Studies, Dalarna University, Falun, Sweden and Visiting Professor at the Institute of Health and Care Sciences, University of Gothenburg, Sweden. PM is a Professor of Healthcare and Nursing Science at the Academic Center for Nursing and Midwifery, Department of Public Health and Primary Care, KU Leuven, Belgium, Visiting Professor at the Institute of Health and Care Sciences, University of Gothenburg, Sweden and Honorary Professor at the Department of Paediatrics and Child Health, University of Cape Town, Cape Town, South Africa. HG is senior lecturer at the Institute of Health and Care Sciences, University of Gothenburg, Sweden and at the University of Gothenburg Centre for Person-Centered Care (GPCC), Sahlgrenska Academy, University of Gothenburg, Gothenburg, Sweden. ELB is senior lecturer at the Institute of Health and Care Sciences, University of Gothenburg, Sweden and a pediatric nurse in Queen Silvia Children's Hospital, Gothenburg, Sweden.

Acknowledgements

For peer review only

We would like to acknowledge and thank the members of the panel of international experts and of the Advisory Board.

For peer review only

References

1. Kennedy A, Sloman F, Douglass JA, et al. Young people with chronic illness: the approach to transition. *Intern Med J*. 2007;37(8):555-60.
2. Mandalenakis Z, Rosengren A, Skoglund K, et al. Survivorship in Children and Young Adults With Congenital Heart Disease in Sweden. *JAMA Intern Med*. 2017;177(2):224-30.
3. Surís JC, Bélanger RE, Ambresin AE, et al. Extra burden of psychosomatic complaints among adolescents suffering from chronic conditions. *J Dev Behav Pediatr*. 2011;32(4):328-31.
4. Suris JC, Michaud PA, Viner R. The adolescent with a chronic condition. Part I: developmental issues. *Arch Dis Child*. 2004;89(10):938-42.
5. Knauth Meadows A, Bosco V, Tong E, et al. Transition and transfer from pediatric to adult care of young adults with complex congenital heart disease. *Curr Cardiol Rep*. 2009;11(4):291-7.
6. Sable C, Foster E, Uzark K, et al. Best practices in managing transition to adulthood for adolescents with congenital heart disease: the transition process and medical and psychosocial issues: a scientific statement from the American Heart Association. *Circulation*. 2011;123(13):1454-85.
7. American Academy of Pediatrics. A consensus statement on health care transitions for young adults with special health care needs. *Pediatrics*. 2002;110(6 Pt 2):1304-6.
8. Warnes CA, Williams RG, Bashore TM, et al. ACC/AHA 2008 guidelines for the management of adults with congenital heart disease: a report of the American College of Cardiology/American Heart Association Task Force on Practice Guidelines (Writing Committee to Develop Guidelines on the Management of Adults With Congenital Heart Disease) Developed in Collaboration With the American Society of Echocardiography, Heart Rhythm Society, International Society for Adult Congenital Heart Disease, Society for Cardiovascular Angiography and Interventions, and Society of Thoracic Surgeons. *J Am Coll Cardiol*. 2008;52(23):E143-E263.
9. Crowley R, Wolfe I, Lock K, et al. Improving the transition between paediatric and adult healthcare: a systematic review. *Arch Dis Child*. 2011;96(6):548-53.
10. Chaturvedi S, Jones CL, Walker RG, et al. The transition of kidney transplant recipients: a work in progress. *Pediatr Nephrol*. 2009;24(5):1055-60.
11. Mackie AS, Islam S, Magill-Evans J, et al. Healthcare transition for youth with heart disease: a clinical trial. *Heart*. 2014;100(14):1113-8.
12. Mackie AS, Rempel GR, Kovacs AH, et al. Transition Intervention for Adolescents With Congenital Heart Disease. *J Am Coll Cardiol*. 2018;71(16):1768-77.
13. Campbell F, Biggs K, Aldiss SK, et al. Transition of care for adolescents from paediatric services to adult health services. *Cochrane Database Syst Rev*. 2016;4:CD009794.
14. James K, Quirk A, Patterson S, et al. Quality of intervention delivery in a cluster randomised controlled trial: a qualitative observational study with lessons for fidelity. *Trials*. 2017;18(1):548.
15. Jeschke KC, Kines P, Rasmussen L, et al. Process evaluation of a Toolbox-training program for construction foremen in Denmark. *Saf Sci*. 2017;94:152-60.
16. Roberts S, McInnes E, Bucknall T, et al. Process evaluation of a cluster-randomised trial testing a pressure ulcer prevention care bundle: a mixed-methods study. *Implement Sci*. 2017;12(1):18.
17. Moore GF, Audrey S, Barker M, et al. Process evaluation of complex interventions: Medical Research Council guidance. *BMJ*. 2015;350:h1258.

For peer review only

18. Bakker FC, Persoon A, Schoon Y, et al. Uniform presentation of process evaluation results facilitates the evaluation of complex interventions: development of a graph. *J Eval Clin Pract*. 2015;21(1):97-102.
19. Hilderson D, Westhovens R, Wouters C, et al. Rationale, design and baseline data of a mixed methods study examining the clinical impact of a brief transition programme for young people with juvenile idiopathic arthritis: the DON'T RETARD project. *BMJ open*. 2013;3(12):e003591.
20. Asp A, Bratt EL, Bramhagen AC. Transfer to Adult Care--Experiences of Young Adults with Congenital Heart Disease. *J Pediatr Nurs*. 2015;30(5):e3-10.
21. Burstrom A, Ojmyr-Joelsson M, Bratt EL, et al. Adolescents With Congenital Heart Disease and Their Parents: Needs Before Transfer to Adult Care. *J Cardiovasc Nurs*. 2016;31(5):399-404.
22. Burström Å, Bratt EL, Frenckner B, et al. Adolescents with congenital heart disease: their opinions about the preparation for transfer to adult care. *Eur J Pediatr*. 2017;176(7):881-89.
23. Bratt EL, Burström Å, Hanseus K, et al. Do not forget the parents—Parents' concerns during transition to adult care for adolescents with congenital heart disease. *Child Care Health Dev*. 2017;44(2):278-84.
24. Bartholomew LK, Markham CM, Ruiter R, et al. Planning health promotion programs: an intervention mapping approach. 4th ed. San Francisco: Jossey-Bass; 2016.
25. Dolk H, Loane M, Garne E, et al. Congenital Heart Defects in EuropeClinical Perspective. *Circulation*. 2011;123(8):841-9.
26. Acuna Mora M, Sparud-Lundin C, Bratt EL, et al. Person-centred transition programme to empower adolescents with congenital heart disease in the transition to adulthood: a study protocol for a hybrid randomised controlled trial (STEPSTONES project). *BMJ Open*. 2017;7(4):e014593.
27. Acuna Mora M, Luyckx K, Sparud-Lundin C, et al. Patient empowerment in young persons with chronic conditions: Psychometric properties of the Gothenburg Young Persons Empowerment Scale (GYPES). *PloS One*. 2018;13(7):e0201007.
28. Small N, Bower P, Chew-Graham CA, et al. Patient empowerment in long-term conditions: development and preliminary testing of a new measure. *BMC Health Serv Res*. 2013;13(1):263.
29. West JF. Public health program planning logic model for community engaged Type 2 diabetes management and prevention. *Eval Program Plann*. 2014;42:43-49.
30. Carroll C, Patterson M, Wood S, et al. A conceptual framework for implementation fidelity. *Implementat Sci*. 2007;2:40.
31. Dobson D, Cook TJ. Avoiding type III error in program evaluation: Results from a field experiment. *Eval Program Plann*. 1980;3(4):269-276.
32. Jansen YJ, Foets MM, de Bont AA. The contribution of qualitative research to the development of tailor-made community-based interventions in primary care: a review. *Eur J Public Health*. 2010 Apr;20(2):220-6.
33. Creswell JW, Plano Clark VL. Designing and conducting mixed methods research. 2nd ed. Los Angeles: SAGE Publications; 2011.
34. Tong A, Sainsbury P, Craig J. Consolidated criteria for reporting qualitative research (COREQ): a 32-item checklist for interviews and focus groups. *Int J Qual Health Care*. 2007;19(6):349-57.
35. Krippendorff K. Content analysis: An introduction to its methodology. Sage; 2012.
36. Wilson AD, Onwuegbuzie AJ, Manning LP. Using paired depth interviews to collect qualitative data. *Qual Rep*. 2016;21(9):1549-73.

For peer review only

37. Griffin TL, Clarke JL, Lancashire ER, et al. Process evaluation results of a cluster randomised controlled childhood obesity prevention trial: The WAVES study. *BMC Public Health*. 2017;17(1):681.
38. Roper J, Shapira J. Ethnography in Nursing Research. London: SAGE Publications Ltd; 2000.
39. Kitzinger J. Qualitative research. Introducing focus groups. *BMJ*. 1995;311(7000):299-302.
40. Graneheim UH, Lundman B. Qualitative content analysis in nursing research: concepts, procedures and measures to achieve trustworthiness. *Nurse Educ Today*. 2004;24(2):105-12.
41. O'Cathain A, Murphy E, Nicholl J. The quality of mixed methods studies in health services research. *J Health Serv Res Policy*. 2008;13(2):92-8.
42. O'Cathain A, Murphy E, Nicholl J. Three techniques for integrating data in mixed methods studies. *BMJ*. 2010;341:c4587.
43. World Medical Association Declaration of Helsinki: ethical principles for medical research involving human subjects. *JAMA*. 2013;310(20):2191-4.
44. Eldh AC, Olai L, Jönsson B, et al. Supporting first-line managers in implementing oral care guidelines in nursing homes. *Nord J Nurs Res*. 2017:2057158517713379.
45. Lloyd J, Dean S, Creanor S, et al. Intervention fidelity in the definitive cluster randomised controlled trial of the Healthy Lifestyles Programme (HeLP) trial: Findings from the process evaluation. *Int J Behav Nutr Phys Act*. 2017;14(1):163.
46. Dusenbury L, Brannigan R, Falco M, et al. A review of research on fidelity of implementation: implications for drug abuse prevention in school settings. *Health Educ Res*. 2003;18(2):237-56.
47. Walton H, Spector A, Tombor I, et al. Measures of fidelity of delivery of, and engagement with, complex, face-to-face health behaviour change interventions: A systematic review of measure quality. *Br J Health Psychol*. 2017;22(4):872-903.
48. Morgan-Trimmer S, Wood F. Ethnographic methods for process evaluations of complex health behaviour interventions. *Trials*. 2016;17(1):232.
49. Steckler AB, Linnan L, Israel B. Process evaluation for public health interventions and research: Jossey-Bass: San Francisco; 2002.
50. Landsberger HA. Hawthorne Revisited: Management and the Worker, Its Critics, and Developments in Human Relations in Industry. 1958.

For peer review only

Figure legends

TC: Transition coordinator

Figure 1: Key components of STEPSTONES transition program and implementation steps

RCT: randomized controlled trial; TC: transition coordinator

Figure 2: Overview of the STEPSTONES RCT-study design

Figure 3: Overview of the process evaluation of the STEPSTONES transition program, adapted from the Medical Research Council's guidance

Figure 4: Overview of STEPSTONES process evaluation components and data sources

For peer review only

Figure 1

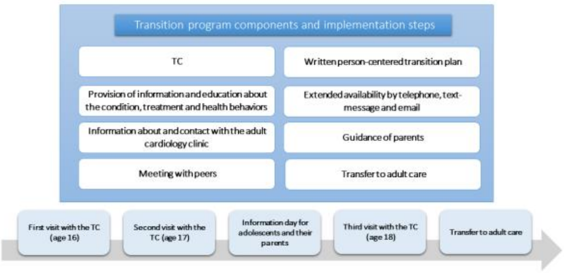

TC: Transition coordinator

Figure 1: Key components of STEPSTONES transition program and implementation steps

For peer review only

Figure 2

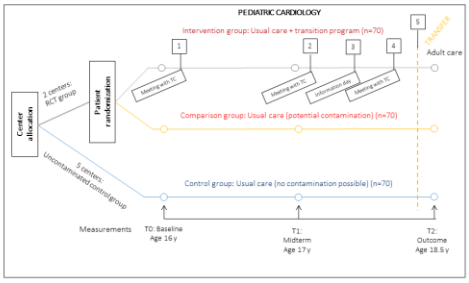

RCT: randomized controlled trial; TC: transition coordinator

Figure 2: Overview of the STEPSTONES RCT-study design

For peer review only

Figure 3

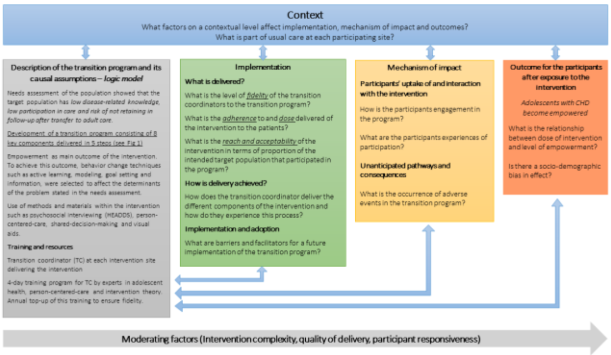

Figure 3: Overview of the process evaluation of the STEPSTONES transition program, adapted from the Medical Research Council's guidance

For peer review only

Figure 4

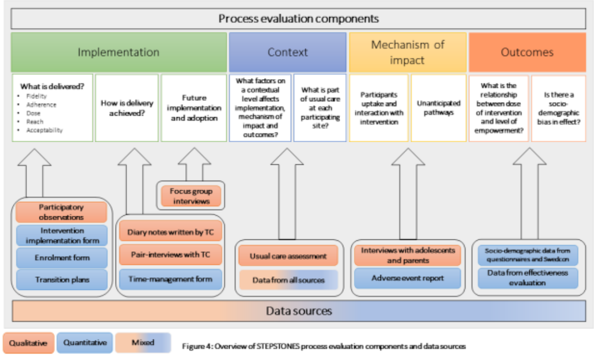

Figure 4: Overview of STEPSTONES process evaluation components and data sources

For peer review only

BMJ Open

Transition program for adolescents with congenital heart disease in transition to adulthood: protocol for a mixed-methods process evaluation study (the STEPSTONES project)

Journal:	*BMJ Open*
Manuscript ID	bmjopen-2018-028229.R1
Article Type:	Protocol
Date Submitted by the Author:	24-Apr-2019
Complete List of Authors:	Saarijärvi, Markus; Goteborgs Universitet, Institute of Health and Care Sciences; KU Leuven, Department of Public Health and Primary Care<br>Wallin, Lars; Hogskolan Dalarna, School of Education, Health and Social studies; Goteborgs Universitet, Institute of Health and Care Sciences<br>Moons, Philip; KU Leuven, Department of Public Health and Primary Care; Goteborgs Universitet, Institute of Health and Care Sciences<br>Gyllensten, Hanna ; Goteborgs Universitet, Institute of Health and Care Sciences; Goteborgs Universitet, University of Gothenburg Centre for Person-Centered Care (GPCC)<br>Bratt, Ewa-Lena; Goteborgs Universitet, Institute of Health and Care Sciences; Drottning Silvias barn- och ungdomssjukhus i Goteborg, Department of Pediatric Cardiology
**Primary Subject Heading**:	Health services research
Secondary Subject Heading:	Research methods, Cardiovascular medicine, Qualitative research
Keywords:	Process evaluation, Randomized controlled trial, Implementation science, Adolescent, Transition to adult care, Heart defect

SCHOLARONE™
Manuscripts

Transition program for adolescents with congenital heart disease in transition to adulthood: protocol for a mixed-methods process evaluation study (the STEPSTONES project)

Markus Saarijärvi MSc, RN[1, 2], Lars Wallin PhD, RN[1, 3-4], Philip Moons PhD, RN[1-2, 5], Hanna Gyllensten PhD, RPH[1, 6], Ewa-Lena Bratt PhD, RN[1, 7]

[1] Institute of Health and Care Sciences, University of Gothenburg, Gothenburg, Sweden

[2] KU Leuven, Department of Public Health and Primary Care, Leuven, Belgium

[3] School of Education, Health and Social Studies, Dalarna University, Falun, Sweden

[4] Department of Neurobiology, Care Sciences and Society, Karolinska Institute, Stockholm, Sweden

[5] Department of Paediatrics and Child Health, University of Cape Town, Cape Town, South Africa

[6] University of Gothenburg Centre for Person-Centered Care (GPCC), Sahlgrenska Academy, University of Gothenburg, Gothenburg, Sweden

[7] Department of Pediatric Cardiology, The Queen Silvia Children's Hospital, Gothenburg, Sweden

Markus Saarijärvi: markus.saarijarvi@gu.se

Lars Wallin: lars.wallin@du.se

Philip Moons: philip.moons@kuleuven.be

Hanna Gyllensten: hanna.gyllensten@gu.se

Ewa-Lena Bratt: ewa-lena.bratt@gu.se

Corresponding author:

Markus Saarijärvi, MSc, RN, Institute of Health and Care Sciences, University of Gothenburg, PO-Box 457, 405 30 Gothenburg, Sweden; Tel: +46 766 61 58, markus.saarijarvi@gu.se

Word count: 3864

For peer review only

ABSTRACT

Introduction

Today, the majority of young persons living with chronic conditions in high-income countries survive into adulthood and will need life-long medical follow-up. Therefore, transition programs have been developed to facilitate transfer to adult care, and to support self-management and independence during adulthood. The STEPSTONES project aims to evaluate the effectiveness of a person-centered transition program for empowering adolescents with congenital heart disease in transition to adulthood. To understand how the transition program causes change and how outcomes are created, process evaluation is imperative to assess implementation, context and mechanisms of impact. This protocol aims to describe the process evaluation of the STEPSTONES transition program.

Methods and design

Medical Research Council guidance for Process evaluation of complex interventions will be the guiding framework for this mixed-methods study. The combination of qualitative and quantitative data will capture different aspects of program delivery. The sample will consist of participants in the STEPSTONES randomized controlled trial (RCT), persons implementing the program, and healthcare professionals. Quantitative data will consist of protocols and routine monitoring documents from the RCT, data collected from patient registries and socio-demographic data to assess the implementation of the intervention. This data will be analyzed with quantitative content analysis, along with descriptive and inferential statistics. Qualitative data will consist of participatory observations, logbooks, and interviews with persons implementing the program, participants and healthcare professionals. Analyses will be performed using qualitative content analysis to investigate mechanism of impact, context and delivery. Quantitative and qualitative data will be integrated in the final stage by using a triangulation protocol according to mixed-methods guidelines.

Ethics and dissemination

The study is approved by the Regional Ethical Review Board in Gothenburg, Sweden. Results will be presented in open-access, peer-reviewed journals and at international scientific conferences.

Keywords

For peer review only - http://bmjopen.bmj.com/site/about/guidelines.xhtml

Adolescent, heart defect, process evaluation, randomized controlled trial, implementation science, transition to adult care.

ARTICLE SUMMARY

Strengths and limitations of this study

- This is the first process evaluation study of a transition program for adolescents with congenital heart disease. Therefore, the study is expected to fill a gap in current knowledge on how transition programs produce change.
- The use of multiple data sources, both quantitative and qualitative, strengthens the quality of the findings by triangulation between data sources.
- Quantitative data in this study rely heavily on routine monitoring documents from the randomized controlled trial, which may affect the trustworthiness of the process evaluation results.
- Process evaluation data will be reported separately from the results from the effectiveness evaluation.

For peer review only

For peer review only - http://bmjopen.bmj.com/site/about/guidelines.xhtml

INTRODUCTION

Today, the majority of young persons living with chronic conditions (CC) in high-income countries survive into adulthood and need life-long follow up.[1, 2] When going from adolescence to adulthood, these young people will face challenges beyond those normally associated with this period in life.[3, 4] Thus, transition programs have been advocated by several guidelines and consensus statements to ensure continuity of care and support self-management and independence in adulthood.[5-7] The current knowledge emphasizes that such programs should be individually tailored, developmentally appropriate and encompass both the physical and psychosocial needs of the adolescent.[8-9] Furthermore, they should include strategies facilitating both transition as a developmental process, and the transfer of care from a pediatric to an adult context.[5] Transition programs have been evaluated to some extent during the last decade, showing improvements in outcomes such as patients' knowledge about their condition, increased self-management, reduced delay in accessing adult care and improved disease-specific indicators.[10-13] However, only four randomized controlled trials (RCT) evaluating transition programs were included in the recent review by the Cochrane Database of Systematic Reviews, and due to the studies lack of sufficient follow-up and rigorous design, the review concluded that existing research on transition programs is insufficient in proving hard evidence on effects.[13] Moreover, none of these RCTs conducted process evaluations with the aim to assess how these interventions created change by assessing the implementation process, contextual factors, and the underlying mechanisms, that led to the outcomes. More evidence is therefore pivotal in explaining the process of care within transition programs to provide knowledge on causality and replicability of these interventions.

Transition programs are complex interventions, comprising numerous interacting ingredients. When the effectiveness of a complex intervention is evaluated, the active ingredients producing the outcome are generally unknown. Process evaluation is therefore indispensable in understanding which these active ingredients are and therefore help explain the mechanism of impact and causal pathways.[14-16] Core elements of process evaluation studies are to investigate how complex interventions (e.g. transition programs) create outcomes, how these outcomes and the change they promote are affected by fidelity and potential moderators on the implementation process, and the impact of contextual factors and participants' engagement. Thus, process evaluation is not solely interested in whether the intended intervention produces effect, but also in unpacking "the black box" of the intervention to investigate how effects are made.[17] In addition to investigating aforementioned aspects, process evaluation studies should

For peer review only - http://bmjopen.bmj.com/site/about/guidelines.xhtml

also explore if the intervention is evaluated properly with regards to internal and external validity of the RCT, and if the intervention can be sustained in practice.[18] Therefore, the STEPSTONES (Swedish Transition Effects Project Supporting Teenagers with chrONic mEdical conditionS) project was established with the purpose of developing, testing and evaluating a person-centered transition program for adolescents with congenital heart disease (CHD). This project will provide new evidence on the effects, causality and replicability of transition programs for adolescents with CCs.

The transition program

The study in focus of the process evaluation is the STEPSTONES project, which is a person-centered transition program for adolescents (age 16-18.5 years) with CHD. The program was developed from a brief transition intervention previously tested in Belgium.[19] To adjust this intervention to the Swedish context, qualitative and quantitative preparatory studies were performed on the target population.[20-23] Furthermore, stakeholder involvement, literature review and behavior change theories were applied, following the protocol for developing health promoting programs, intervention mapping.[24] The program aims to be generic, and thus applicable to other CCs. However, CHD was chosen as the target population of this intervention, being the most common congenital malformation in need of life-long follow up.[25] The program consists of eight key components, implemented in five steps (see Fig. 1). A transition coordinator (TC) operating at each intervention center delivers the program. The TC is a specialized, pediatric nurse who has received tailored training in delivering the intervention.

Participants and setting – RCT-study

The STEPSTONES program is evaluated through a hybrid RCT, where a longitudinal observational study is embedded within the RCT (Fig. 2). The study is performed in seven University hospitals in Sweden where two centers consist of an intervention group (n=70), and a comparison group (n=70) where individual randomization is performed to either of the groups. The remaining five centers constitute additional control groups (n=70) where no contamination of the intervention is expected. The hypothesis is that adolescents randomized to receive the transition program over a two-year period will have a higher patient empowerment score compared to those randomized to usual care. Inclusion criteria for participants in the RCT-study are: 16 years of age with CHD, Swedish speaking and literate. Their parents will also be invited to participate. The study is ongoing and will be concluded in 2021. A study protocol describing the RCT has been published.[26]

For peer review only - http://bmjopen.bmj.com/site/about/guidelines.xhtml

Outcome measures of the intervention

The primary outcome of patient empowerment is measured using the GYPES (Gothenburg Young Persons Empowerment Scale).[27] Patient empowerment is defined as "an enabling process or outcome arising from communication with the healthcare professional and a mutual sharing of resources over information relating to illness, which enhances the patient's feelings of control, self-efficacy, coping abilities and ability to achieve change over their condition".[28] The secondary outcomes are transition readiness, disease-related knowledge, health behaviors, patient-reported health, quality of life, healthcare usage and parental uncertainty towards transfer to adult care.[26] The process evaluation is focused around understanding how patient empowerment is achieved within the transition program and how delivery of the intervention, participants' engagement and contextual factors impacts in achieving this outcome.

Process evaluation of the transition program

In accordance with the Medical Research Council's (MRC) guidance on process evaluation of complex interventions, key aspects of this evaluation are centered on the logic model of the intervention, the implementation, mechanism of impact and context.[17] These aspects have a direct impact on how effectively interventions work in producing the desired outcome. The logic model of the intervention provides a structure for designing, carrying out and analyzing process data.[17, 29] This model can also work as a blueprint of the intervention, describing the underlying theoretical assumptions, input (e.g. theory, training and resources), how the intervention is intended to be delivered, components of the intervention, and how the intervention aims to cause change.[17] In Fig. 3, the logic model of the program (grey box) is presented along with the key concepts in process evaluation according to the MRC guidance.[17] This figure provides an overview, starting from the logic model, on how the process evaluation of the STEPSTONES transition program is structured.

The concept of implementation within this model focuses on how successful delivery of the intervention is accomplished and what is actually being delivered (i.e. fidelity). Fidelity is described as "the degree to which the clinical intervention was delivered as it was intended" and is assessed by evaluating adherence, dose and reach of each component of the intervention.[30] Fidelity may have a direct impact on outcomes, and thus researchers cannot determine if an intervention failed due to poor implementation or to inadequacies in the program, unless fidelity is measured.[31]

For peer review only - http://bmjopen.bmj.com/site/about/guidelines.xhtml

The concept of mechanism of impact refers to how the intervention activities produce intended or unintended effects,[17] in this case the main outcome of patient empowerment. This can be studied by including data on participants' responsiveness and interaction with the intervention, their trajectory of care within the intervention, and by assessing possible moderators such as quality of delivery and complexity of the intervention.[17, 30]

Finally, context includes factors external to the intervention, which influences implementation, or whether the mechanisms of impact act as intended within the context in which the intervention is being delivered.[17] Contextual factors have shown to impact how the intervention is adapted locally and in addition, shape the outcomes of the intervention.[32]

By exploring the relationship between the logic model, how the intervention is delivered in practice and relating this to the potential moderators of delivery of an intervention (such as fidelity and context), the outcomes of the intervention can be understood.[30] Therefore, the overall aim is to perform a process evaluation study, assessing the implementation of the program and the contextual impact on this process in order to understand and explain the mechanisms creating the outcome of the transition program. are listed together with proposed research methods in Fig. 4.

METHOD

A mixed-methods embedded design will be used, including collection, analysis and integration of quantitative and qualitative data.[33] During the designing and planning of the data collection the process evaluation guidance by the MRC[14] was chosen as the methodological framework.

Participants and setting

For the quantitative assessment, participants (n=70) and participating parents in the intervention group will be included. In addition, the TCs at the intervention centers, as well as a sample of physicians and nurses at all participating centers will be asked to participate. For the qualitative assessment, we will use a purposive sub-sample according to qualitative methodological guidelines. [34] Specifically, we will strive for variability of the participants' characteristics in order to capture a breadth of experiences.

Data collection

For peer review only - http://bmjopen.bmj.com/site/about/guidelines.xhtml

Data will consist of a range of sources in order to capture different perspectives of key process variables. Quantitative and qualitative data collection will be carried out simultaneously and synthesized in the final analysis.

Quantitative data collection

The following data sources will be used to collect quantitative data on process measures:

1) *Enrolment form*
    - The enrolment form is based on the five delivery steps of the intervention (see Fig. 1). The data allows for measurement of adherence and attendance in the intervention for all eligible participants. Drop out from the study, non-attendance for visits and reasons for this will also be registered, which gives information about acceptability of the transition program.
2) *Intervention implementation form*
    - This form will be filled out by the TC after each individual patient encounter, whether in person, by telephone, text message or email. This form contains the components of the intervention, e.g. information and education about the health condition, treatment and health behavior, and information about the adult care follow-up. It also gives information about topics covered, supportive activities and recommendations given, providing an indication about adherence, and dose delivered of the intervention to the patients. The data source will also give information on which communication channel is primarily used for contact between the TC and patients.
3) *Adverse event report (AE)*
    - The occurrence of AE's will give an indication of potential attrition from the study.
4) *Transition plans*
    - Each patient in the intervention receives a transition plan in which the care is documented. An analysis of this plan will be undertaken to understand what the main objectives were in the transition of individual patients. This analysis will estimate which components of the intervention have been delivered, the outcomes of these, and how this is documented. The data from these sources will give information about possible mechanism of impact, adherence, dose and the potential effects of moderating factors on fidelity such as patient characteristics.
5) *Socio-demographic data*

For peer review only - http://bmjopen.bmj.com/site/about/guidelines.xhtml

- Age, sex, education level, diagnosis and pharmacotherapy, comorbidities, geographic location, living situation, country of birth, parental marital status and employment situation will be retrieved from the Swedish registry of congenital heart disease (Swedcon) and the questionnaires used in the RCT. This data will be used to examine the characteristics of participants who engage with and continue participation in the intervention, and those who do not (e.g. drop-outs and non-participants), and how this affects the reach of the program.

6) *Time-management form*
   - In this form, the TCs register how much time is spent on performing different tasks of the transition program, such as preparation of visits, patient contacts, administration and contacts with other healthcare professionals. Time spent on study-related tasks (e.g. recruitment, randomization and data-entry) is excluded from this form. By collecting data on time-management, differences in intervention delivery between the two centers will be visible, also giving valuable information to a future implementation scenario.

Qualitative data collection

The qualitative data will be used to answer questions on how implementation is achieved, possible moderating factors and how the participants experience and engage with the intervention. Qualitative data will be collected and analyzed by a researcher (MS) that is not involved in the RCT-study, to ensure trustworthiness of the data.[17] The following qualitative data sources will be used:

1) *Pair-interviews with TC*
   - Throughout the course of the trial, pair-interviews[35] with the TCs will be performed every six months. An interview guide will be used covering topics such as the experiences of implementing the intervention, barriers and facilitators and local contextual factors.
2) *Diary notes written by the TC*
   - Reflective diary notes are requested from the TCs, describing their experiences of implementing the transition program, barriers and facilitators and contextual factors they perceive to have affected the intervention.[36]
3) *Participatory observations*
   - Observations of outpatient visits will be performed to investigate how the transition program is being implemented in practice and the responsiveness and

For peer review only - http://bmjopen.bmj.com/site/about/guidelines.xhtml

engagement of the participants. The observations will provide data on several process variables such as fidelity, adaptations, dose and participants engagement. Both a structured observation protocol and field notes will be used. The protocol includes components from the intervention implementation form and in addition, practical applications of how the intervention is intended to be delivered, e.g. use of materials and methods to deliver the behavior change techniques and application of person-centered-care. Informal interviews will be conducted with the TCs and participants in connection with the observations as a way of ensuring trustworthiness.[37]

- Observations of an information day for adolescents and their parents, will be held between the second and third outpatient visit in the transition program. During the information day, adolescents and their parents will get the opportunity to meet their peers, receive information about the adult care and discuss issues regarding transfer and transition. Participatory observations of these meetings will be performed, including informal interviews with the participants about their experiences of the day.

4) *Semi-structured interviews with adolescents and parents*
   - Participants and their parents will be interviewed about their experiences one month after completion of the transition program. The interviews will give increased understanding of the dose received by the participants, potential moderating factors on intervention delivery, acceptability of the intervention and potential improvements for a future implementation of the transition program into current practice.

5) *Focus group interviews with the healthcare team*
   - Focus group interviews will be held with interdisciplinary teams from the intervention centers. The aim is to explore what contextual factors on a local and organizational level are perceived to affect implementation and what prerequisites that are needed for a future implementation of the transition program. The sample will consist of physicians and nurses from both pediatric cardiology and adult care with experiences of young persons with CHD.[38]

6) *Usual care assessment*
   - Throughout the course of the trial, assessment of the usual care in the control and comparison groups will be undertaken through participatory observations of outpatient visits and interviews with healthcare professionals. The objective is

For peer review only - http://bmjopen.bmj.com/site/about/guidelines.xhtml

to assess current practices, barriers and facilitators for future implementation and identify possible contamination between study sites.

Data analysis

Quantitative data analysis

The process variables (e.g. adherence and dose) retrieved from the enrolment form, intervention implementation form and socio-demographic data will be entered into a database and analyzed in the Statistical Package for the Social Sciences (SPSS) by using descriptive statistics with frequency distributions, proportions, variability over time and means. The effect on process variables of intervention centers' characteristics and patients' socio-demographic factors will be analyzed by regression analysis, as will data from the time-management form, to explain potential moderators on fidelity. The reach of the intervention (i.e. did the intended population participate in the study), will be assessed by comparing participants versus non-participants of the RCT with socio-demographic data and clinical data (e.g. disease characteristics). The MRC guidance recommends reporting results of the process evaluation separately from the effectiveness evaluation to avoid biased interpretation of effectiveness results.[17] We are following this recommendation but will in addition perform a post-hoc regression analysis on dose-outcome relationships as well as sub-group analysis for relevant clinical and demographical variables.

The data from the transition plans will be analyzed using descriptive statistics along with quantitative content analysis [39] using the software NVivo (QSR International Pty Ltd. Version 12, 2018) by counting word occurrences related to the components of the intervention to summarize what the main objectives were of each individual participant's transition. Adverse events will be analyzed if they occur.

Qualitative data analysis

Design, sampling, analysis and reporting of the qualitative findings will follow the Consolidated Criteria for Reporting Qualitative research.[34] Qualitative data will be analyzed with manifest content analysis, as described by Graneheim and Lundman.[40] All qualitative data analysis will be performed in the software NVivo.

Integrating quantitative and qualitative data

For peer review only - http://bmjopen.bmj.com/site/about/guidelines.xhtml

Analysis and reporting of the mixed-method study will follow the Good Reporting of A Mixed Methods Study framework.[41] The mix of quantitative and qualitative data can give information about contextual factors that affect implementation and potential moderating factors on fidelity.[17] Qualitative data can be used to explain quantitative findings.[33] The integration of data will be performed by creating a triangulation protocol, which works as a scheme where the different data sources are combined and findings from different components of the process evaluation are listed and compared. By looking for agreement and disagreement between the data sources the findings may lead to a better understanding of the results.[42]

Patient and public involvement

The STEPSTONES project was developed with the support of an advisory board and panel of international experts consisting of stakeholders, i.e. young adults with CHD, parents, clinicians, researchers and representatives from the patient organization. Patients will not be involved in the recruitment of this study; however, young adults with CHD will be involved during the adolescent day sharing their experiences of the transfer to adult care and transition to adulthood, acting as role models and providing peer support. Dissemination of results to study participants will be delivered through the patient organization and social media.

Ethics and dissemination

Adolescents, parents and health care professionals in the RCT have consented to participation after receiving written and oral information about the study. Written consent to participate in the qualitative data collection will be requested after additional written and oral information about the study. Ethical approval for the study was received from the Regional Ethical Review Board in Gothenburg, Sweden (No.931-15), and the study will be performed in accordance to the Declaration of Helsinki.[43] Findings of the process evaluation study will be published in open-access, peer-reviewed scientific journals and presented at national and international conferences.

DISCUSSION

Process evaluation studies have been increasing in recent years and are imperative to explaining how complex interventions produce the desired outcomes. Furthermore, such evaluation studies are arguably more important when interventions fail to produce the intended outcomes. Since its release in 2015, MRC guidance has been used in process evaluation studies, providing

For peer review only - http://bmjopen.bmj.com/site/about/guidelines.xhtml

extensive data on important aspects of interventions delivery.[44-45] However, MRC does lack certain potentially important aspects, for example, the impact of recruitment and the internal and external validity of the RCT on outcomes. For this reason, we have added these research questions to our protocol.

The proposed study has several strengths. Firstly, when implementing a complex intervention, a clear description of the logic model of the intervention is beneficial, not only as a benchmark for evaluators but also for those delivering the intervention. Previous studies show that complex interventions with clear descriptions have a greater chance of being implemented with high adherence.[46] The logic model of our intervention was developed using the framework of intervention mapping, which provides a thorough evidence base.[24] In addition, the materials, resources and training for the TCs can facilitate strategies, enhancing intervention fidelity.[30] This process evaluation study can thus add to the existing evidence on how frameworks, such as intervention mapping, can aid in intervention development, implementation and evaluation.[24]

Secondly, the planned extent of data sources, methods and scope of data collection have, to the best of our knowledge, not been applied in previous process evaluation studies. This is beneficial for many reasons. The use of various sources can strengthen understanding of how different factors interact in creating outcomes. A recent review concluded that only one quarter of all studies measuring fidelity and engagement in interventions used multiple data collection methods such as observations and self-reported measures.[47] A range of qualitative methods within intervention studies can therefore strengthen the evaluation since these methods are forceful in capturing contextual factors.[32, 48] Moreover, quantitative data provides measures for important process variables such as fidelity.[30] By collecting data throughout the trial, this study can also handle the impact of 'teething problems' (e.g. TCs learning curves) by following changes over time and combining quantitative measures with stakeholder interviews.[17]

Thirdly, the findings of the process evaluation will be reported separately from the effectiveness evaluation of the STEPSTONES study, avoiding biased interpretations of results and therefore strengthening the validity of the findings.[17] Since the process measures of the study rely on routine monitoring data, validity of these documents has to be ensured by quality control and cross-checking between different sources. Routine monitoring data are effective in reducing response bias; they are cost-effective and allow for analysis over time.[17]

For peer review only - http://bmjopen.bmj.com/site/about/guidelines.xhtml

However, some challenges and limitations of this process evaluation study have to be considered. Factors affecting the recruitment are not directly included in the qualitative data collection. In Steckler and Linnan's[49] framework, this is a part of the process evaluation which is not included in the MRC guidance.[17] Recruitment pace, selection of study participants and recruiters' skills could arguably be considered moderating factors on reach and outcomes. That said, the acceptability of the intervention is explored and measured, and so is reach in terms of participants approached and recruited. Furthermore, the TCs performing the intervention, and their performance during the intervention, will have a great impact on many of the key process measures since they are the only personnel delivering the intervention. To manage this, data will also be collected from participants and healthcare professionals in order to broaden the perspective. Process evaluators should also be aware of how they impact the quality of the collected data (e.g. Hawthorne-effects), especially in participatory observations.[50] However, a small sub-sample of qualitative observations from a total of 210 outpatient visits should arguably give little or no impact on effectiveness outcomes of the RCT. Nevertheless, it may affect the quality of the process evaluation results. To manage this, establishing good relations between the researchers performing the process evaluation and the TCs and healthcare professionals is important to get access to the field and retrieve trustworthy data. Informal interviews in connection with observations can also strengthen trustworthiness.[48]

Conclusions

Transfer of care for adolescents living with chronic conditions is a timely issue, and the effectiveness and causal assumptions of transition programs are yet to be proven. It is therefore imperative to conduct high-quality process evaluation studies to understand how the programs are delivered, for whom they are delivered and under what circumstances. The effect of contextual factors and possible moderating factors on intervention fidelity and participant engagement may also increase transferability of findings and can thus inform implementation of transition programs in other settings.

List of abbreviations

AE: Adverse Event

CHD: Congenital Heart Disease

MRC: Medical Research Council

For peer review only

For peer review only - http://bmjopen.bmj.com/site/about/guidelines.xhtml

RCT: Randomized Controlled Trial

STEPSTONES: Swedish Transition Effects Project Supporting Teenagers with chronic mEdical conditionS

SWEDCON: Swedish congenital heart disease registry

TC: Transition coordinator

Competing interests' statement

The authors declare that they have no competing interest.

Funding statement

This work was supported by research grants from the Swedish Heart-Lung Foundation (grant 20150535); Swedish Research Council for Health, Working Life and Welfare-FORTE (grant STYA-2015/0003); Swedish Children Heart Association; Swedish Research Council (grant 2015-02503); and the Institute of Health and Care Sciences of the University of Gothenburg.

Author contributions

MS, LW, PM, HG and ELB conceived the study and were responsible for the design and draft of the study protocol. All authors critically revised and approved the manuscript.

Author information

MS is a PhD student at the Institute of Health and Care Sciences, University of Gothenburg, Sweden and at the Academic Center for Nursing and Midwifery, Department of Public Health and Primary Care, KU Leuven, Belgium. LW is a Professor of Nursing Science at the School of Education, Health and Social Studies, Dalarna University, Falun, Sweden and Visiting Professor at the Institute of Health and Care Sciences, University of Gothenburg, Sweden. PM is a Professor of Healthcare and Nursing Science at the Academic Center for Nursing and Midwifery, Department of Public Health and Primary Care, KU Leuven, Belgium, Visiting Professor at the Institute of Health and Care Sciences, University of Gothenburg, Sweden and Honorary Professor at the Department of Paediatrics and Child Health, University of Cape Town, Cape Town, South Africa. HG is senior lecturer at the Institute of Health and Care Sciences, University of Gothenburg, Sweden and at the University of Gothenburg Centre for Person-Centered Care (GPCC), Sahlgrenska Academy, University of Gothenburg, Gothenburg,

For peer review only - http://bmjopen.bmj.com/site/about/guidelines.xhtml

Sweden. ELB is senior lecturer at the Institute of Health and Care Sciences, University of Gothenburg, Sweden and a pediatric nurse in Queen Silvia Children's Hospital, Gothenburg, Sweden.

Acknowledgements

We would like to acknowledge and thank the members of the panel of international experts and of the Advisory Board.

For peer review only

For peer review only - http://bmjopen.bmj.com/site/about/guidelines.xhtml

References

1. Kennedy A, Sloman F, Douglass JA, et al. Young people with chronic illness: the approach to transition. *Intern Med J*. 2007;37(8):555-60.
2. Mandalenakis Z, Rosengren A, Skoglund K, et al. Survivorship in Children and Young Adults With Congenital Heart Disease in Sweden. *JAMA Intern Med*. 2017;177(2):224-30.
3. Surís JC, Bélanger RE, Ambresin AE, et al. Extra burden of psychosomatic complaints among adolescents suffering from chronic conditions. *J Dev Behav Pediatr*. 2011;32(4):328-31.
4. Suris JC, Michaud PA, Viner R. The adolescent with a chronic condition. Part I: developmental issues. *Arch Dis Child*. 2004;89(10):938-42.
5. Knauth Meadows A, Bosco V, Tong E, et al. Transition and transfer from pediatric to adult care of young adults with complex congenital heart disease. *Curr Cardiol Rep*. 2009;11(4):291-7.
6. Sable C, Foster E, Uzark K, et al. Best practices in managing transition to adulthood for adolescents with congenital heart disease: the transition process and medical and psychosocial issues: a scientific statement from the American Heart Association. *Circulation*. 2011;123(13):1454-85.
7. American Academy of Pediatrics. A consensus statement on health care transitions for young adults with special health care needs. *Pediatrics*. 2002;110(6 Pt 2):1304-6.
8. Warnes CA, Williams RG, Bashore TM, et al. ACC/AHA 2008 guidelines for the management of adults with congenital heart disease: a report of the American College of Cardiology/American Heart Association Task Force on Practice Guidelines (Writing Committee to Develop Guidelines on the Management of Adults With Congenital Heart Disease) Developed in Collaboration With the American Society of Echocardiography, Heart Rhythm Society, International Society for Adult Congenital Heart Disease, Society for Cardiovascular Angiography and Interventions, and Society of Thoracic Surgeons. *J Am Coll Cardiol*. 2008;52(23):E143-E263.
9. Crowley R, Wolfe I, Lock K, et al. Improving the transition between paediatric and adult healthcare: a systematic review. *Arch Dis Child*. 2011;96(6):548-53.
10. Chaturvedi S, Jones CL, Walker RG, et al. The transition of kidney transplant recipients: a work in progress. *Pediatr Nephrol*. 2009;24(5):1055-60.
11. Mackie AS, Islam S, Magill-Evans J, et al. Healthcare transition for youth with heart disease: a clinical trial. *Heart*. 2014;100(14):1113-8.
12. Mackie AS, Rempel GR, Kovacs AH, et al. Transition Intervention for Adolescents With Congenital Heart Disease. *J Am Coll Cardiol*. 2018;71(16):1768-77.
13. Campbell F, Biggs K, Aldiss SK, et al. Transition of care for adolescents from paediatric services to adult health services. *Cochrane Database Syst Rev*. 2016;4:CD009794.
14. James K, Quirk A, Patterson S, et al. Quality of intervention delivery in a cluster randomised controlled trial: a qualitative observational study with lessons for fidelity. *Trials*. 2017;18(1):548.
15. Jeschke KC, Kines P, Rasmussen L, et al. Process evaluation of a Toolbox-training program for construction foremen in Denmark. *Saf Sci*. 2017;94:152-60.
16. Roberts S, McInnes E, Bucknall T, et al. Process evaluation of a cluster-randomised trial testing a pressure ulcer prevention care bundle: a mixed-methods study. *Implement Sci*. 2017;12(1):18.
17. Moore GF, Audrey S, Barker M, et al. Process evaluation of complex interventions: Medical Research Council guidance. *BMJ*. 2015;350:h1258.

For peer review only - http://bmjopen.bmj.com/site/about/guidelines.xhtml

18. Bakker FC, Persoon A, Schoon Y, et al. Uniform presentation of process evaluation results facilitates the evaluation of complex interventions: development of a graph. *J Eval Clin Pract*. 2015;21(1):97-102.
19. Hilderson D, Westhovens R, Wouters C, et al. Rationale, design and baseline data of a mixed methods study examining the clinical impact of a brief transition programme for young people with juvenile idiopathic arthritis: the DON'T RETARD project. *BMJ open*. 2013;3(12):e003591.
20. Asp A, Bratt EL, Bramhagen AC. Transfer to Adult Care--Experiences of Young Adults with Congenital Heart Disease. *J Pediatr Nurs*. 2015;30(5):e3-10.
21. Burstrom A, Ojmyr-Joelsson M, Bratt EL, et al. Adolescents With Congenital Heart Disease and Their Parents: Needs Before Transfer to Adult Care. *J Cardiovasc Nurs*. 2016;31(5):399-404.
22. Burström Å, Bratt EL, Frenckner B, et al. Adolescents with congenital heart disease: their opinions about the preparation for transfer to adult care. *Eur J Pediatr*. 2017;176(7):881-89.
23. Bratt EL, Burström Å, Hanseus K, et al. Do not forget the parents—Parents' concerns during transition to adult care for adolescents with congenital heart disease. *Child Care Health Dev*. 2017;44(2):278-84.
24. Bartholomew LK, Markham CM, Ruiter R, et al. Planning health promotion programs: an intervention mapping approach. 4th ed. San Francisco: Jossey-Bass; 2016.
25. Dolk H, Loane M, Garne E, et al. Congenital Heart Defects in EuropeClinical Perspective. *Circulation*. 2011;123(8):841-9.
26. Acuna Mora M, Sparud-Lundin C, Bratt EL, et al. Person-centred transition programme to empower adolescents with congenital heart disease in the transition to adulthood: a study protocol for a hybrid randomised controlled trial (STEPSTONES project). *BMJ Open*. 2017;7(4):e014593.
27. Acuna Mora M, Luyckx K, Sparud-Lundin C, et al. Patient empowerment in young persons with chronic conditions: Psychometric properties of the Gothenburg Young Persons Empowerment Scale (GYPES). *PloS One*. 2018;13(7):e0201007.
28. Small N, Bower P, Chew-Graham CA, et al. Patient empowerment in long-term conditions: development and preliminary testing of a new measure. *BMC Health Serv Res*. 2013;13(1):263.
29. West JF. Public health program planning logic model for community engaged Type 2 diabetes management and prevention. *Eval Program Plann*. 2014;42:43-49.
30. Carroll C, Patterson M, Wood S, et al. A conceptual framework for implementation fidelity. *Implementat Sci*. 2007;2:40.
31. Dobson D, Cook TJ. Avoiding type III error in program evaluation: Results from a field experiment. *Eval Program Plann*. 1980;3(4):269-276.
32. Jansen YJ, Foets MM, de Bont AA. The contribution of qualitative research to the development of tailor-made community-based interventions in primary care: a review. *Eur J Public Health*. 2010 Apr;20(2):220-6.
33. Creswell JW, Plano Clark VL. Designing and conducting mixed methods research. 2nd ed. Los Angeles: SAGE Publications; 2011.
34. Tong A, Sainsbury P, Craig J. Consolidated criteria for reporting qualitative research (COREQ): a 32-item checklist for interviews and focus groups. *Int J Qual Health Care*. 2007;19(6):349-57.
35. Wilson AD, Onwuegbuzie AJ, Manning LP. Using paired depth interviews to collect qualitative data. *Qual Rep*. 2016;21(9):1549-73.

For peer review only - http://bmjopen.bmj.com/site/about/guidelines.xhtml

36. Griffin TL, Clarke JL, Lancashire ER, et al. Process evaluation results of a cluster randomised controlled childhood obesity prevention trial: The WAVES study. *BMC Public Health*. 2017;17(1):681.
37. Roper J, Shapira J. Ethnography in Nursing Research. London: SAGE Publications Ltd; 2000.
38. Kitzinger J. Qualitative research. Introducing focus groups. *BMJ*. 1995;311(7000):299-302.
39. Krippendorff K. Content analysis: An introduction to its methodology. Sage; 2012.
40. Graneheim UH, Lundman B. Qualitative content analysis in nursing research: concepts, procedures and measures to achieve trustworthiness. *Nurse Educ Today.* 2004;24(2):105-12.
41. O'Cathain A, Murphy E, Nicholl J. The quality of mixed methods studies in health services research. *J Health Serv Res Policy*. 2008;13(2):92-8.
42. O'Cathain A, Murphy E, Nicholl J. Three techniques for integrating data in mixed methods studies. *BMJ*. 2010;341:c4587.
43. World Medical Association Declaration of Helsinki: ethical principles for medical research involving human subjects. *JAMA*. 2013;310(20):2191-4.
44. Eldh AC, Olai L, Jönsson B, et al. Supporting first-line managers in implementing oral care guidelines in nursing homes. *Nord J Nurs Res.* 2017:2057158517713379.
45. Lloyd J, Dean S, Creanor S, et al. Intervention fidelity in the definitive cluster randomised controlled trial of the Healthy Lifestyles Programme (HeLP) trial: Findings from the process evaluation. *Int J Behav Nutr Phys Act*. 2017;14(1):163.
46. Dusenbury L, Brannigan R, Falco M, et al. A review of research on fidelity of implementation: implications for drug abuse prevention in school settings. *Health Educ Res*. 2003;18(2):237-56.
47. Walton H, Spector A, Tombor I, et al. Measures of fidelity of delivery of, and engagement with, complex, face-to-face health behaviour change interventions: A systematic review of measure quality. *Br J Health Psychol*. 2017;22(4):872-903.
48. Morgan-Trimmer S, Wood F. Ethnographic methods for process evaluations of complex health behaviour interventions. *Trials*. 2016;17(1):232.
49. Steckler AB, Linnan L, Israel B. Process evaluation for public health interventions and research: Jossey-Bass: San Francisco; 2002.
50. Landsberger HA. Hawthorne Revisited: Management and the Worker, Its Critics, and Developments in Human Relations in Industry. 1958.

For peer review only

For peer review only - http://bmjopen.bmj.com/site/about/guidelines.xhtml

Figure legends

TC: Transition coordinator

Figure 1: Key components of STEPSTONES transition program and implementation steps

RCT: randomized controlled trial; TC: transition coordinator

Figure 2: Overview of the STEPSTONES RCT-study design

Figure 3: Overview of the process evaluation of the STEPSTONES transition program, adapted from the Medical Research Council's guidance

Figure 4: Overview of STEPSTONES process evaluation components and data sources

For peer review only

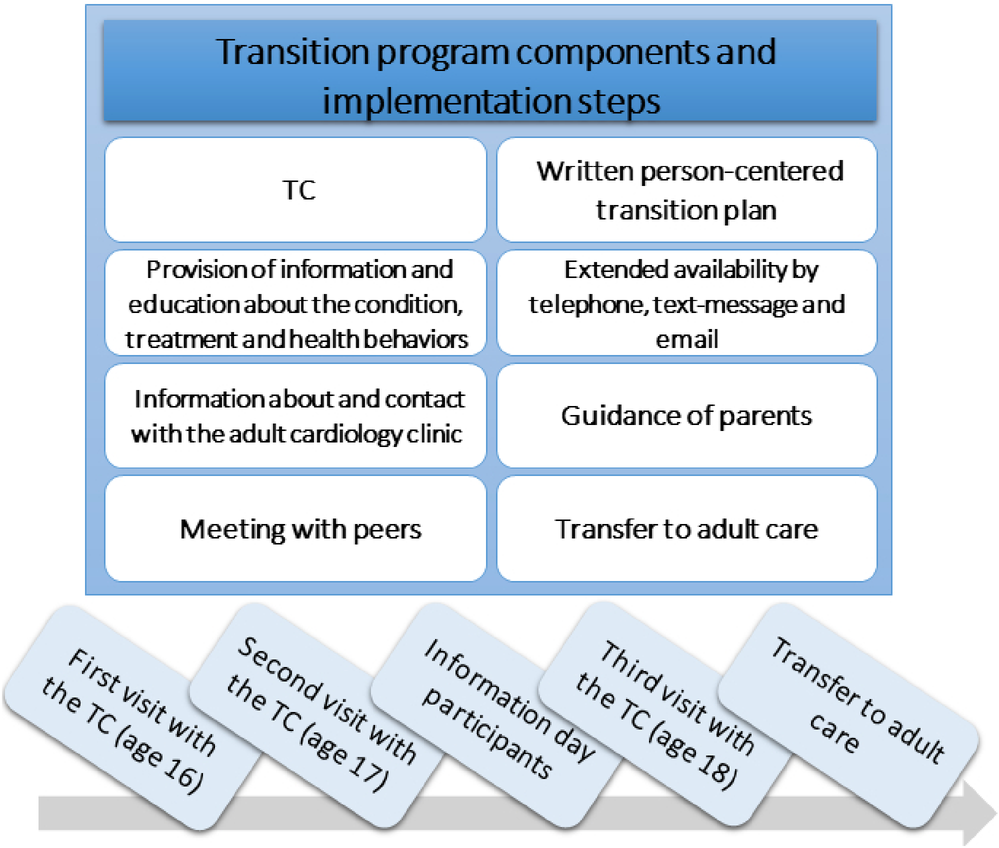

TC: Transition coordinator
Figure 1: Key components of STEPSTONES transition program and implementation steps

58x50mm (300 x 300 DPI)

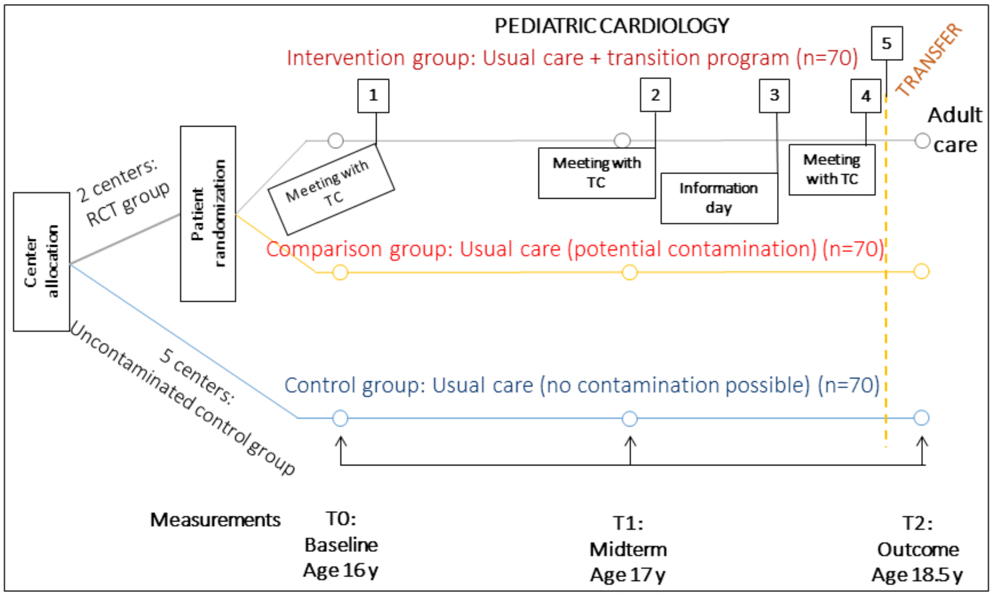

RCT: randomized controlled trial
Figure 2: Overview of the STEPSTONES RCT-study design

73x44mm (300 x 300 DPI)

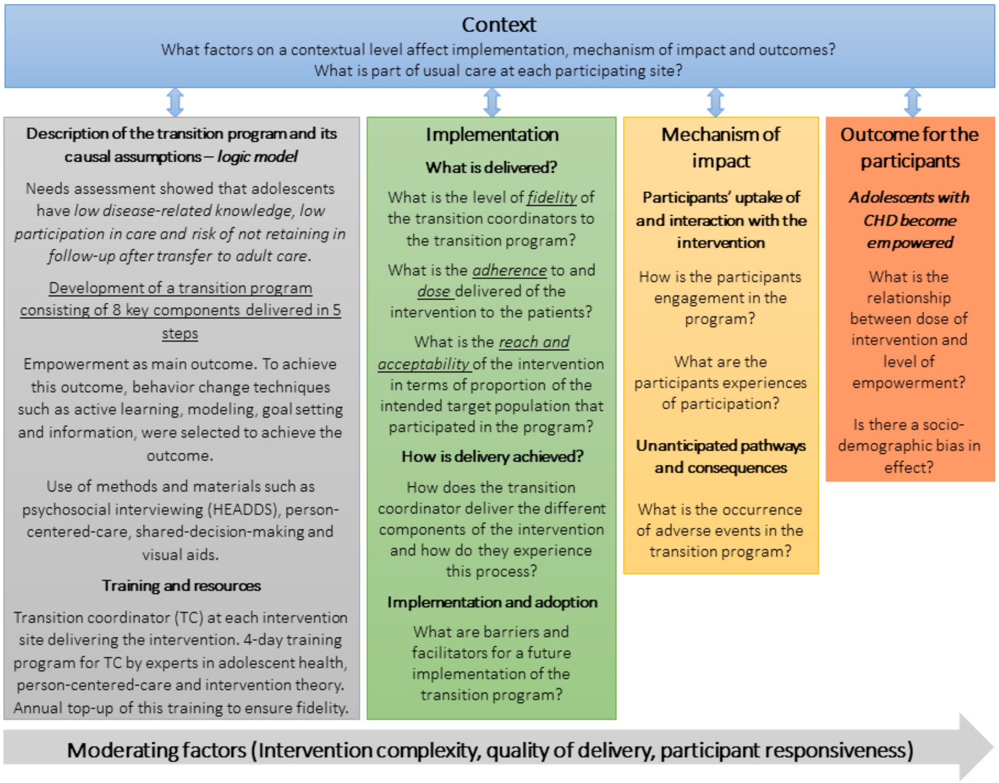

Figure 3: Overview of the process evaluation of the STEPSTONES transition program, adapted from the Medical Research Council's guidance

76x60mm (300 x 300 DPI)

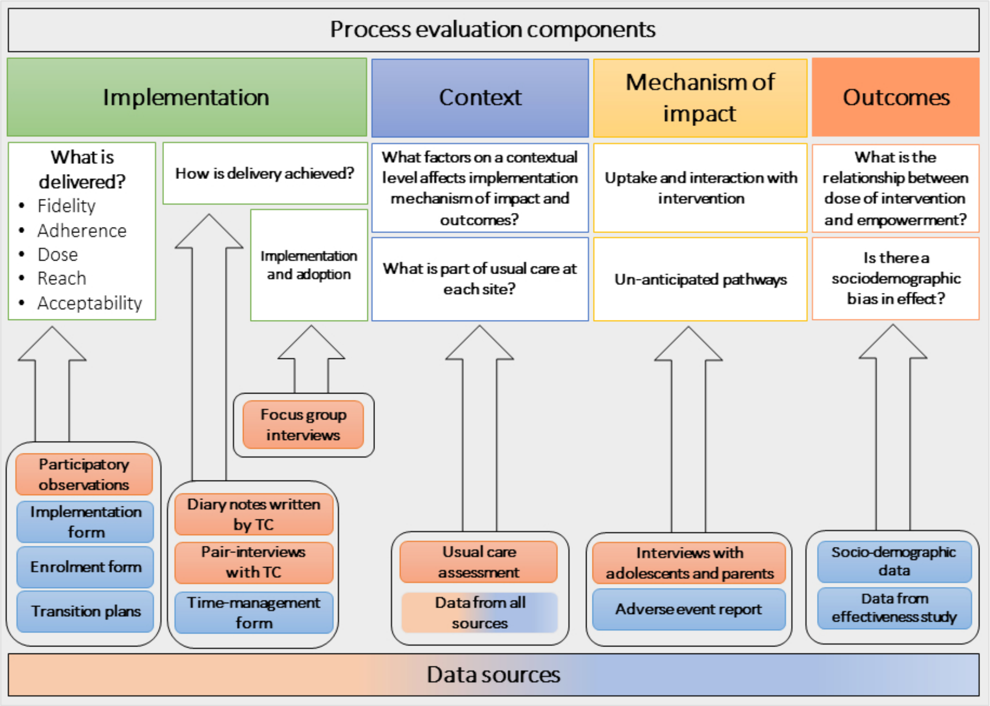

Figure 4: Overview of STEPSTONES process evaluation components and data sources

78x55mm (300 x 300 DPI)

BMJ Open

Transition program for adolescents with congenital heart disease in transition to adulthood: protocol for a mixed-methods process evaluation study (the STEPSTONES project)

Journal:	*BMJ Open*
Manuscript ID	bmjopen-2018-028229.R2
Article Type:	Protocol
Date Submitted by the Author:	03-Jul-2019
Complete List of Authors:	Saarijärvi, Markus; Goteborgs Universitet, Institute of Health and Care Sciences; KU Leuven, Department of Public Health and Primary Care<br>Wallin, Lars; Hogskolan Dalarna, School of Education, Health and Social studies; Goteborgs Universitet, Institute of Health and Care Sciences<br>Moons, Philip; KU Leuven, Department of Public Health and Primary Care; Goteborgs Universitet, Institute of Health and Care Sciences<br>Gyllensten, Hanna ; Goteborgs Universitet, Institute of Health and Care Sciences; Goteborgs Universitet, University of Gothenburg Centre for Person-Centered Care (GPCC)<br>Bratt, Ewa-Lena; Goteborgs Universitet, Institute of Health and Care Sciences; Drottning Silvias barn- och ungdomssjukhus i Goteborg, Department of Pediatric Cardiology
**Primary Subject Heading**:	Health services research
Secondary Subject Heading:	Research methods, Cardiovascular medicine, Qualitative research, Paediatrics
Keywords:	Process evaluation, Randomized controlled trial, Implementation science, Adolescent, Transition to adult care, Heart defect

SCHOLARONE™
Manuscripts

Transition program for adolescents with congenital heart disease in transition to adulthood: protocol for a mixed-methods process evaluation study (the STEPSTONES project)

Markus Saarijärvi MSc, RN[1, 2], Lars Wallin PhD, RN[1, 3-4], Philip Moons PhD, RN[1-2, 5], Hanna Gyllensten PhD, RPH[1, 6], Ewa-Lena Bratt PhD, RN[1, 7]

[1] Institute of Health and Care Sciences, University of Gothenburg, Gothenburg, Sweden

[2] KU Leuven, Department of Public Health and Primary Care, Leuven, Belgium

[3] School of Education, Health and Social Studies, Dalarna University, Falun, Sweden

[4] Department of Neurobiology, Care Sciences and Society, Karolinska Institute, Stockholm, Sweden

[5] Department of Paediatrics and Child Health, University of Cape Town, Cape Town, South Africa

[6] University of Gothenburg Centre for Person-Centered Care (GPCC), Sahlgrenska Academy, University of Gothenburg, Gothenburg, Sweden

[7] Department of Pediatric Cardiology, The Queen Silvia Children's Hospital, Gothenburg, Sweden

Markus Saarijärvi: markus.saarijarvi@gu.se

Lars Wallin: lars.wallin@du.se

Philip Moons: philip.moons@kuleuven.be

Hanna Gyllensten: hanna.gyllensten@gu.se

Ewa-Lena Bratt: ewa-lena.bratt@gu.se

Corresponding author:

Markus Saarijärvi, MSc, RN, Institute of Health and Care Sciences, University of Gothenburg, PO-Box 457, 405 30 Gothenburg, Sweden; Tel: +46 766 61 58, markus.saarijarvi@gu.se

Word count: 4095

For peer review only

ABSTRACT

Introduction

Today, the majority of young persons living with chronic conditions in high-income countries survive into adulthood and will need life-long medical follow-up. Therefore, transition programs have been developed to facilitate transfer to adult care, and to support self-management and independence during adulthood. The STEPSTONES project aims to evaluate the effectiveness of a person-centered transition program for empowering adolescents with congenital heart disease in transition to adulthood. To understand how the transition program causes change and how outcomes are created, process evaluation is imperative to assess implementation, context and mechanisms of impact. This protocol aims to describe the process evaluation of the STEPSTONES transition program.

Methods and design

Medical Research Council guidance for Process evaluation of complex interventions will be the guiding framework for this mixed-methods study. The combination of qualitative and quantitative data will capture different aspects of program delivery. The sample will consist of participants in the STEPSTONES randomized controlled trial (RCT), persons implementing the program, and healthcare professionals. Quantitative data will consist of protocols and routine monitoring documents from the RCT, data collected from patient registries and socio-demographic data to assess the implementation of the intervention. This data will be analyzed with quantitative content analysis, along with descriptive and inferential statistics. Qualitative data will consist of participatory observations, logbooks, and interviews with persons implementing the program, participants and healthcare professionals. Analyses will be performed using qualitative content analysis to investigate mechanism of impact, context and delivery. Quantitative and qualitative data will be integrated in the final stage by using a triangulation protocol according to mixed-methods guidelines.

Ethics and dissemination

The study is approved by the Regional Ethical Review Board in Gothenburg, Sweden. Results will be presented in open-access, peer-reviewed journals and at international scientific conferences.

Keywords

For peer review only - http://bmjopen.bmj.com/site/about/guidelines.xhtml

Adolescent, heart defect, process evaluation, randomized controlled trial, implementation science, transition to adult care.

ARTICLE SUMMARY

Strengths and limitations of this study

- This is the first process evaluation study of a transition program for adolescents with congenital heart disease. Therefore, the study is expected to fill a gap in current knowledge on how transition programs produce change.
- The use of multiple data sources, both quantitative and qualitative, strengthens the quality of the findings by triangulation between data sources.
- Quantitative data in this study rely heavily on routine monitoring documents from the randomized controlled trial, which may affect the trustworthiness of the process evaluation results.
- Process evaluation data will be reported separately from the results from the effectiveness evaluation.

For peer review only

For peer review only - http://bmjopen.bmj.com/site/about/guidelines.xhtml

INTRODUCTION

Today, the majority of young persons living with chronic conditions (CC) in high-income countries survive into adulthood and need life-long follow up.[1, 2] When going from adolescence to adulthood, these young people will face challenges beyond those normally associated with this period in life.[3, 4] Thus, transition programs have been advocated by several guidelines and consensus statements to ensure continuity of care and support self-management and independence in adulthood.[5-7] The current knowledge emphasizes that such programs should be individually tailored, developmentally appropriate and encompass both the physical and psychosocial needs of the adolescent.[8-9] Furthermore, they should include strategies facilitating both transition as a developmental process, and the transfer of care from a pediatric to an adult context.[5] Transition programs have been evaluated to some extent during the last decade, showing improvements in outcomes such as patients' knowledge about their condition, increased self-management, reduced delay in accessing adult care and improved disease-specific indicators.[10-13] However, only four randomized controlled trials (RCT) evaluating transition programs were included in the recent review by the Cochrane Database of Systematic Reviews, and due to the studies lack of sufficient follow-up and rigorous design, the review concluded that existing research on transition programs is insufficient in proving hard evidence on effects.[13] Moreover, none of these RCTs conducted process evaluations with the aim to assess how these interventions created change by assessing the implementation process, contextual factors, and the underlying mechanisms, that led to the outcomes. More evidence is therefore pivotal in explaining the process of care within transition programs to provide knowledge on causality and replicability of these interventions.

Transition programs are complex interventions, comprising numerous interacting ingredients. When the effectiveness of a complex intervention is evaluated, the active ingredients producing the outcome are generally unknown. Process evaluation is therefore indispensable in understanding which these active ingredients are and therefore help explain the mechanism of impact and causal pathways.[14-16] Core elements of process evaluation studies are to investigate how complex interventions (e.g. transition programs) create outcomes, how these outcomes and the change they promote are affected by fidelity and potential moderators on the implementation process, and the impact of contextual factors and participants' engagement. Thus, process evaluation is not solely interested in whether the intended intervention produces effect, but also in unpacking "the black box" of the intervention to investigate how effects are made.[17] In addition to investigating aforementioned aspects, process evaluation studies should

For peer review only - http://bmjopen.bmj.com/site/about/guidelines.xhtml

also explore if the intervention is evaluated properly with regards to internal and external validity of the RCT, and if the intervention can be sustained in practice.[18] Therefore, the STEPSTONES (Swedish Transition Effects Project Supporting Teenagers with chrONic mEdical conditionS) project was established with the purpose of developing, testing and evaluating a person-centered transition program for adolescents with congenital heart disease (CHD). This project will provide new evidence on the effects, causality and replicability of transition programs for adolescents with CCs.

The transition program

The study in focus of the process evaluation reported here is the STEPSTONES project, which is a person-centered transition program for adolescents (age 16-18.5 years) with CHD. The program was developed from a brief transition intervention previously tested in Belgium.[19] To adjust this intervention to the Swedish context, qualitative and quantitative preparatory studies were performed on the target population.[20-23] Furthermore, stakeholder involvement, literature review and behavior change theories were applied, following the protocol for developing health promoting programs, intervention mapping.[24] The program aims to be generic, and thus applicable to other CCs. However, CHD was chosen as the target population of this intervention, being the most common congenital malformation in need of life-long follow up.[25] The program consists of eight key components, implemented in five steps (see Fig. 1). A transition coordinator (TC) operating at each intervention center delivers the program. The TC is a specialized, pediatric nurse who has received tailored training in delivering the intervention.

Participants and setting – RCT-study

The STEPSTONES program is evaluated through a hybrid RCT, where a longitudinal observational study is embedded within the RCT (Fig. 2). The study is performed in seven University hospitals in Sweden where two centers consist of an intervention group (n=70), and a comparison group (n=70) where individual randomization is performed to either of the groups. The remaining five centers constitute additional control groups (n=70) where no contamination of the intervention is expected. The hypothesis is that adolescents randomized to receive the transition program over a two-year period will have a higher patient empowerment score compared to those randomized to usual care. Inclusion criteria for participants in the RCT-study are: 16 years of age with CHD, Swedish speaking and literate. Their parents will also be invited to participate. The study is ongoing and will be concluded in 2021. A study protocol describing the RCT has been published.[26]The process evaluation of the STEPSTONES project is

For peer review only - http://bmjopen.bmj.com/site/about/guidelines.xhtml

conducted alongside the effectiveness- and health economic evaluation in order to understand and explain the outcomes of the trial. Since the process evaluation is extensive, covering several different aspects of program delivery, the full extent of this evaluation is reported in this paper.

Outcome measures of the intervention

The primary outcome of patient empowerment is measured using the GYPES (Gothenburg Young Persons Empowerment Scale). GYPES has been psychometrically tested and proven to be valid for the study population.[27] Patient empowerment is defined as "an enabling process or outcome arising from communication with the healthcare professional and a mutual sharing of resources over information relating to illness, which enhances the patient's feelings of control, self-efficacy, coping abilities and ability to achieve change over their condition".[28] The secondary outcomes are transition readiness, disease-related knowledge, health behaviors, patient-reported health, quality of life, healthcare usage and parental uncertainty towards transfer to adult care.[26] The process evaluation is focused on understanding how patient empowerment is achieved within the transition program and how delivery of the intervention, participants' engagement and contextual factors impacts in achieving this outcome.

Process evaluation of the transition program

In accordance with the Medical Research Council's (MRC) guidance on process evaluation of complex interventions, key aspects of this evaluation are centered on the logic model of the intervention, the implementation, mechanism of impact and context.[17] These aspects have a direct impact on how effectively interventions work in producing the desired outcome. The logic model of the intervention provides a structure for designing, carrying out and analyzing process data.[17, 29] This model can also work as a blueprint of the intervention, describing the underlying theoretical assumptions, input (e.g. theory, training and resources), how the intervention is intended to be delivered, components of the intervention, and how the intervention aims to cause change.[17] In Fig. 3, the logic model of the STEPSTONES transition program is presented along with the key concepts in process evaluation according to the MRC guidance.[17] This figure provides an overview, starting from the logic model, on how the process evaluation of the STEPSTONES transition program is structured.

The concept of implementation within this model focuses on how successful delivery of the intervention is accomplished and what is actually being delivered (i.e. fidelity). Fidelity is described as "the degree to which the clinical intervention was delivered as it was intended"

For peer review only - http://bmjopen.bmj.com/site/about/guidelines.xhtml

and is assessed by evaluating adherence, dose and reach of each component of the intervention.[30] Fidelity may have a direct impact on outcomes, and thus researchers cannot determine if an intervention failed due to poor implementation or to inadequacies in the program, unless fidelity is measured.[31] In addition, it is important to assess the process of delivering the intervention. For instance, exploring barriers and facilitators as expressed by the TCs, and how potential moderating factors (e.g. quality of delivery) affects how the program is delivered. [17, 30]

The concept of mechanism of impact refers to how the activities performed within the intervention produce intended or unintended effects,[17] in this case the main outcome of patient empowerment. The mechanism of impact for the participants from the intervention will be explored by assessing observable actions of patient empowerment. According to the program theory, this can include if adolescents have developed the necessary skills to become the manager of their health and care, if they are engaged in the learning process about their health and care, and if they remain in follow up after the transfer to adult care. The mechanism of impact can be studied by including data on participants' responsiveness and interaction with the intervention, their trajectory of care, and by assessing possible moderators such as quality of delivery and complexity of the intervention.[17, 30]

Finally, context includes factors external to the intervention, which influences implementation, or whether the mechanisms of impact act as intended within the context in which the intervention is being delivered.[17] Contextual factors have shown to impact how the intervention is adapted locally and in addition, shape the outcomes of the intervention.[32] Therefore, the overall aim is to perform a process evaluation study, assessing the implementation of the program and the contextual impact on this process in order to understand and explain the mechanisms creating the outcome of the transition program. The research questions in relation to the key process evaluation concepts are listed together with proposed research methods in Fig. 4.

METHOD

A mixed-methods embedded design will be used, including collection, analysis and integration of quantitative and qualitative data.[33] During the designing and planning of the data collection the process evaluation guidance by the MRC[14] was chosen as the methodological framework.

For peer review only - http://bmjopen.bmj.com/site/about/guidelines.xhtml

Participants and setting

For the quantitative assessment, participants (n=70) and participating parents in the intervention group will be included. In addition, the TCs at the intervention centers, as well as a sample of physicians and nurses at all participating centers will be asked to participate. For the qualitative assessment, we will use a purposive sub-sample since it is not considered feasible to include all participants in the intervention group (n=70) in the qualitative data collection. Specifically, we will strive for variability of the participants' characteristics in order to capture a breadth of experiences. [34]

Data collection

Data will consist of a range of sources in order to capture different perspectives of key process variables. Quantitative and qualitative data collection will be carried out simultaneously and synthesized in the final analysis.

Quantitative data collection

The following data sources will be used to collect quantitative data on process measures:

1) *Enrolment form*
   - The enrolment form is based on the five delivery steps of the intervention (see Fig. 1). The data allows for measurement of adherence and attendance in the intervention for all eligible participants. Drop out from the study, non-attendance for visits and reasons for this will also be registered, which gives information about acceptability of the transition program.
2) *Intervention implementation form*
   - This form will be filled out by the TC after each individual patient encounter, whether in person, by telephone, text message or email. This form contains the components of the intervention, e.g. information and education about the health condition, treatment and health behavior, and information about the adult care follow-up. It also gives information about topics covered, supportive activities and recommendations given, providing an indication about adherence, and dose delivered of the intervention to the patients. The data source will also give information on which communication channel is primarily used for contact between the TC and patients.
3) *Adverse event report (AE)*

For peer review only - http://bmjopen.bmj.com/site/about/guidelines.xhtml

- The occurrence of AE's will give an indication of potential attrition from the study.

4) *Transition plans*
   - Each patient in the intervention receives a transition plan in which the care is documented. An analysis of this plan will be undertaken to understand what the main objectives were in the transition of individual patients. This analysis will estimate which components of the intervention have been delivered, the outcomes of these, and how this is documented. The data from these sources will give information about possible mechanism of impact, adherence, dose and the potential effects of moderating factors on fidelity such as patient characteristics.
5) *Socio-demographic data*
   - Age, sex, education level, diagnosis and pharmacotherapy, comorbidities, geographic location, living situation, country of birth, parental marital status and employment situation will be retrieved from the Swedish registry of congenital heart disease (Swedcon) and the questionnaires used in the RCT. This data will be used to examine the characteristics of participants who engage with and continue participation in the intervention, and those who do not (e.g. drop-outs and non-participants), and how this affects the reach of the program.
6) *Time-management form*
   - In this form, the TCs register how much time is spent on performing different tasks of the transition program, such as preparation of visits, patient contacts, administration and contacts with other healthcare professionals. Time spent on study-related tasks (e.g. recruitment, randomization and data-entry) is excluded from this form. By collecting data on time-management, differences in intervention delivery between the two centers will be visible, also giving valuable information to a future implementation scenario.

Qualitative data collection

The qualitative data will be used to answer questions on how implementation is achieved, possible moderating factors and how the participants experience and engage with the intervention. Qualitative data will be collected and analyzed by a researcher (MS) that is not involved in the RCT-study, to ensure trustworthiness of the data.[17] The following qualitative data sources will be used:

1) *Pair-interviews with TC*

For peer review only - http://bmjopen.bmj.com/site/about/guidelines.xhtml

- Throughout the course of the trial, pair-interviews[35] with the TCs will be performed every six months. An interview guide will be used covering topics such as the experiences of implementing the intervention, barriers and facilitators and local contextual factors.

2) *Diary notes written by the TC*
   - Reflective diary notes are requested from the TCs, describing their experiences of implementing the transition program, barriers and facilitators and contextual factors they perceive to have affected the intervention.[36]

3) *Participatory observations*
   - Observations of outpatient visits will be performed to investigate how the transition program is being implemented in practice and the responsiveness and engagement of the participants. The observations will provide data on several process variables such as fidelity, adaptations, dose and participants engagement. Both a structured observation protocol and field notes will be used. The protocol includes components from the intervention implementation form and in addition, practical applications of how the intervention is intended to be delivered, e.g. use of materials and methods to deliver the behavior change techniques and application of person-centered-care. Informal interviews will be conducted with the TCs and participants in connection with the observations as a way of ensuring trustworthiness.[37]
   - Observations of an information day for adolescents and their parents, will be held between the second and third outpatient visit in the transition program. During the information day, adolescents and their parents will get the opportunity to meet their peers, receive information about the adult care and discuss issues regarding transfer and transition. Participatory observations of these meetings will be performed, including informal interviews with the participants about their experiences of the day.

4) *Semi-structured interviews with adolescents and parents*
   - Participants and their parents will be interviewed about their experiences one month after completion of the transition program. The interviews will give increased understanding of the dose received by the participants, potential moderating factors on intervention delivery, acceptability of the intervention and potential improvements for a future implementation of the transition program into current practice.

For peer review only - http://bmjopen.bmj.com/site/about/guidelines.xhtml

5) *Focus group interviews with the healthcare team*
   - Focus group interviews will be held with interdisciplinary teams from the intervention centers. The aim is to explore what contextual factors on a local and organizational level are perceived to affect implementation and what prerequisites that are needed for a future implementation of the transition program. The sample will consist of physicians and nurses from both pediatric cardiology and adult care with experiences of young persons with CHD.[38]

6) *Usual care assessment*
   - Throughout the course of the trial, assessment of the usual care in the control and comparison groups will be undertaken through participatory observations of outpatient visits and interviews with healthcare professionals. The objective is to assess current practices, barriers and facilitators for future implementation and identify possible contamination between study sites.

Data analysis

Quantitative data analysis

The process variables (e.g. adherence and dose) retrieved from the enrolment form, intervention implementation form and socio-demographic data will be entered into a database and analyzed in the Statistical Package for the Social Sciences (SPSS) by using descriptive statistics with frequency distributions, proportions, variability over time and means. The effect on process variables of intervention centers' characteristics and patients' socio-demographic factors will be analyzed by regression analysis, as will data from the time-management form, to explain potential moderators on fidelity. The reach of the intervention (i.e. did the intended population participate in the study), will be assessed by comparing participants versus non-participants of the RCT with socio-demographic data and clinical data (e.g. disease characteristics). The MRC guidance recommends reporting results of the process evaluation separately from the effectiveness evaluation to avoid biased interpretation of effectiveness results.[17] We are following this recommendation but will in addition perform a post-hoc regression analysis on dose-outcome relationships as well as sub-group analysis for relevant clinical and demographical variables.

The data from the transition plans will be analyzed using descriptive statistics along with quantitative content analysis[39] using the software NVivo (QSR International Pty Ltd. Version 12, 2018) by counting word occurrences related to the components of the intervention to

For peer review only - http://bmjopen.bmj.com/site/about/guidelines.xhtml

summarize what the main objectives were of each individual participant's transition. Adverse events will be analyzed if they occur.

Qualitative data analysis

Design, sampling, analysis and reporting of the qualitative findings will follow the Consolidated Criteria for Reporting Qualitative research.[40] Qualitative data will be analyzed with manifest content analysis, as described by Graneheim and Lundman. [41] The MRC guidance for process evaluation will act as a coding matrix for the qualitative data in the analysis phase. [17] All qualitative data analysis will be performed in the software NVivo.

Integrating quantitative and qualitative data

Analysis and reporting of the mixed-method study will follow the Good Reporting of A Mixed Methods Study framework.[42] The mix of quantitative and qualitative data can give information about contextual factors that affect implementation and potential moderating factors on fidelity.[17] Qualitative data can be used to explain quantitative findings.[33] The integration of data will be performed by creating a triangulation protocol, which works as a scheme where the different data sources are combined and findings from different components of the process evaluation are listed and compared. By looking for agreement and disagreement between the data sources the findings may lead to a better understanding of the results.[43]

Patient and public involvement

The STEPSTONES project was developed with the support of an advisory board and panel of international experts consisting of stakeholders, i.e. young adults with CHD, parents, clinicians, researchers and representatives from the patient organization. Patients will not be involved in the recruitment of this study; however, young adults with CHD will be involved during the adolescent day sharing their experiences of the transfer to adult care and transition to adulthood, acting as role models and providing peer support. Dissemination of results to study participants will be delivered through the patient organization and social media.

Ethics and dissemination

Adolescents, parents and health care professionals in the RCT have consented to participation after receiving written and oral information about the study. Written consent to participate in the qualitative data collection will be requested after additional written and oral information about the study. Ethical approval for the study was received from the Regional Ethical Review

For peer review only - http://bmjopen.bmj.com/site/about/guidelines.xhtml

Board in Gothenburg, Sweden (No.931-15), and the study will be performed in accordance to the Declaration of Helsinki.[44] Findings of the process evaluation study will be published in open-access, peer-reviewed scientific journals and presented at national and international conferences.

DISCUSSION

Process evaluation studies have been increasing in recent years and are imperative to explaining how complex interventions produce the desired outcomes. Furthermore, such evaluation studies are arguably more important when interventions fail to produce the intended outcomes. Since its release in 2015, MRC guidance has been used in process evaluation studies, providing extensive data on important aspects of interventions delivery.[45-46] However, MRC does lack certain potentially important aspects, for example, the impact of recruitment and the internal and external validity of the RCT on outcomes. For this reason, we have added these research questions to our protocol.

The proposed study has several strengths. Firstly, solid funding of the process evaluation has allowed us to employ adequate number of research staff to handle logistics regarding recruitment, data collection and analysis. Furthermore, documentation of process variables is digital and can be followed up continuously from the main research site.

Secondly, when implementing a complex intervention, a clear description of the intervention is beneficial, not only as a benchmark for evaluators but also for those delivering the intervention. Previous studies show that complex interventions with clear descriptions have a greater chance of being implemented with high adherence.[47] The logic model of our intervention was developed using the framework of intervention mapping, which provides a thorough evidence base.[24] In addition, the materials, resources and training for the TCs can facilitate delivery, enhancing intervention fidelity.[30] This process evaluation study can thus add to the existing evidence on how frameworks, such as intervention mapping, can aid in intervention development and evaluation.[24]

Thirdly, the planned extent of data sources, methods and scope of data collection have, to the best of our knowledge, not been applied in previous process evaluation studies. This is beneficial for many reasons. The use of various sources can strengthen understanding of how different factors interact in creating outcomes. A recent review concluded that only one quarter

For peer review only - http://bmjopen.bmj.com/site/about/guidelines.xhtml

of all studies measuring fidelity and engagement in interventions used multiple data collection methods such as observations and self-reported measures.[48] A range of qualitative methods within intervention studies can therefore strengthen the evaluation since these methods are forceful in capturing contextual factors.[32, 49] Moreover, quantitative data provides measures for important process variables such as fidelity.[30] By collecting data throughout the trial, this study can also handle the impact of 'teething problems' (e.g. TCs learning curves) by following changes over time and combining quantitative measures with stakeholder interviews.[17]

Finally, the findings of the process evaluation will be reported separately from the effectiveness evaluation, avoiding biased interpretations of results and therefore strengthening the validity of the findings.[17] Since the process measures in thisstudy rely on routine monitoring data, validity of these documents has to be ensured by quality control and cross-checking between different sources. Routine monitoring data are effective in reducing response bias; they are cost-effective and allow for analysis over time.[17]

However, some challenges and limitations of this process evaluation study have to be considered. Factors affecting recruitment are not directly included in the qualitative data collection. In Steckler and Linnan's[50] framework, this is a part of the process evaluation which is not included in the MRC guidance.[17] Recruitment pace, selection of study participants and recruiters' skills could arguably be considered moderating factors on reach and outcomes. Moreover, the considerable amount of questionnaires distributed to participants in the effectiveness evaluation might affect willingness to participate and therefore adherence. That said, the acceptability of the intervention is explored and measured, and so is reach in terms of participants approached and recruited. Furthermore, the TCs performing the intervention, and their performance during the intervention, will have a great impact on many of the key process measures since they are the only personnel delivering the intervention. To manage this, data will also be collected from participants and healthcare professionals in order to broaden the perspective. Process evaluators should also be aware of how they impact the quality of the collected data (e.g. Hawthorne-effects), especially in participatory observations.[51] However, a small sub-sample of qualitative observations from a total of 210 outpatient visits should arguably give little or no impact on effectiveness outcomes of the RCT. Nevertheless, it may affect the quality of the process evaluation results. To manage this, establishing good relations between the researchers performing the process evaluation and the TCs and healthcare

For peer review only - http://bmjopen.bmj.com/site/about/guidelines.xhtml

professionals is important to get access to the field and retrieve trustworthy data. Informal interviews in connection with observations can also strengthen trustworthiness.[49]

Conclusions

Transfer of care for adolescents living with chronic conditions is a timely issue, and the effectiveness and causal assumptions of transition programs are yet to be proven. It is therefore imperative to conduct high-quality process evaluation studies to understand how the programs are delivered, for whom they are delivered and under what circumstances. The effect of contextual factors and possible moderating factors on intervention fidelity and participant engagement may also increase transferability of findings and can thus inform implementation of transition programs in other settings.

List of abbreviations

AE: Adverse Event

CHD: Congenital Heart Disease

MRC: Medical Research Council

RCT: Randomized Controlled Trial

STEPSTONES: Swedish Transition Effects Project Supporting Teenagers with chronic mEdical conditionS

SWEDCON: Swedish congenital heart disease registry

TC: Transition coordinator

Competing interests' statement

The authors declare that they have no competing interest.

Funding statement

This work was supported by research grants from the Swedish Heart-Lung Foundation (grant 20150535); Swedish Research Council for Health, Working Life and Welfare-FORTE (grant

For peer review only - http://bmjopen.bmj.com/site/about/guidelines.xhtml

For peer review only

STYA-2015/0003); Swedish Children Heart Association; Swedish Research Council (grant 2015-02503); and the Institute of Health and Care Sciences of the University of Gothenburg.

Author contributions

MS, LW, PM, HG and ELB conceived the study and were responsible for the design and draft of the study protocol. All authors critically revised and approved the manuscript.

Author information

MS is a PhD student at the Institute of Health and Care Sciences, University of Gothenburg, Sweden and at the Academic Center for Nursing and Midwifery, Department of Public Health and Primary Care, KU Leuven, Belgium. LW is a Professor of Nursing Science at the School of Education, Health and Social Studies, Dalarna University, Falun, Sweden and Visiting Professor at the Institute of Health and Care Sciences, University of Gothenburg, Sweden. PM is a Professor of Healthcare and Nursing Science at the Academic Center for Nursing and Midwifery, Department of Public Health and Primary Care, KU Leuven, Belgium, Visiting Professor at the Institute of Health and Care Sciences, University of Gothenburg, Sweden and Honorary Professor at the Department of Paediatrics and Child Health, University of Cape Town, Cape Town, South Africa. HG is senior lecturer at the Institute of Health and Care Sciences, University of Gothenburg, Sweden and at the University of Gothenburg Centre for Person-Centered Care (GPCC), Sahlgrenska Academy, University of Gothenburg, Gothenburg, Sweden. ELB is senior lecturer at the Institute of Health and Care Sciences, University of Gothenburg, Sweden and a pediatric nurse in Queen Silvia Children's Hospital, Gothenburg, Sweden.

Acknowledgements

We would like to acknowledge and thank the members of the panel of international experts and of the Advisory Board.

For peer review only

References

1. Kennedy A, Sloman F, Douglass JA, et al. Young people with chronic illness: the approach to transition. *Intern Med J*. 2007;37(8):555-60.
2. Mandalenakis Z, Rosengren A, Skoglund K, et al. Survivorship in Children and Young Adults With Congenital Heart Disease in Sweden. *JAMA Intern Med*. 2017;177(2):224-30.
3. Surís JC, Bélanger RE, Ambresin AE, et al. Extra burden of psychosomatic complaints among adolescents suffering from chronic conditions. *J Dev Behav Pediatr*. 2011;32(4):328-31.
4. Suris JC, Michaud PA, Viner R. The adolescent with a chronic condition. Part I: developmental issues. *Arch Dis Child*. 2004;89(10):938-42.
5. Knauth Meadows A, Bosco V, Tong E, et al. Transition and transfer from pediatric to adult care of young adults with complex congenital heart disease. *Curr Cardiol Rep*. 2009;11(4):291-7.
6. Sable C, Foster E, Uzark K, et al. Best practices in managing transition to adulthood for adolescents with congenital heart disease: the transition process and medical and psychosocial issues: a scientific statement from the American Heart Association. *Circulation*. 2011;123(13):1454-85.
7. American Academy of Pediatrics. A consensus statement on health care transitions for young adults with special health care needs. *Pediatrics*. 2002;110(6 Pt 2):1304-6.
8. Warnes CA, Williams RG, Bashore TM, et al. ACC/AHA 2008 guidelines for the management of adults with congenital heart disease: a report of the American College of Cardiology/American Heart Association Task Force on Practice Guidelines (Writing Committee to Develop Guidelines on the Management of Adults With Congenital Heart Disease) Developed in Collaboration With the American Society of Echocardiography, Heart Rhythm Society, International Society for Adult Congenital Heart Disease, Society for Cardiovascular Angiography and Interventions, and Society of Thoracic Surgeons. *J Am Coll Cardiol.* 2008;52(23):E143-E263.
9. Crowley R, Wolfe I, Lock K, et al. Improving the transition between paediatric and adult healthcare: a systematic review. *Arch Dis Child*. 2011;96(6):548-53.
10. Chaturvedi S, Jones CL, Walker RG, et al. The transition of kidney transplant recipients: a work in progress. *Pediatr Nephrol*. 2009;24(5):1055-60.
11. Mackie AS, Islam S, Magill-Evans J, et al. Healthcare transition for youth with heart disease: a clinical trial. *Heart*. 2014;100(14):1113-8.
12. Mackie AS, Rempel GR, Kovacs AH, et al. Transition Intervention for Adolescents With Congenital Heart Disease. *J Am Coll Cardiol*. 2018;71(16):1768-77.
13. Campbell F, Biggs K, Aldiss SK, et al. Transition of care for adolescents from paediatric services to adult health services. *Cochrane Database Syst Rev*. 2016;4:CD009794.
14. James K, Quirk A, Patterson S, et al. Quality of intervention delivery in a cluster randomised controlled trial: a qualitative observational study with lessons for fidelity. *Trials*. 2017;18(1):548.
15. Jeschke KC, Kines P, Rasmussen L, et al. Process evaluation of a Toolbox-training program for construction foremen in Denmark. *Saf Sci*. 2017;94:152-60.
16. Roberts S, McInnes E, Bucknall T, et al. Process evaluation of a cluster-randomised trial testing a pressure ulcer prevention care bundle: a mixed-methods study. *Implement Sci*. 2017;12(1):18.
17. Moore GF, Audrey S, Barker M, et al. Process evaluation of complex interventions: Medical Research Council guidance. *BMJ*. 2015;350:h1258.

For peer review only - http://bmjopen.bmj.com/site/about/guidelines.xhtml

18. Bakker FC, Persoon A, Schoon Y, et al. Uniform presentation of process evaluation results facilitates the evaluation of complex interventions: development of a graph. *J Eval Clin Pract*. 2015;21(1):97-102.
19. Hilderson D, Westhovens R, Wouters C, et al. Rationale, design and baseline data of a mixed methods study examining the clinical impact of a brief transition programme for young people with juvenile idiopathic arthritis: the DON'T RETARD project. *BMJ open*. 2013;3(12):e003591.
20. Asp A, Bratt EL, Bramhagen AC. Transfer to Adult Care--Experiences of Young Adults with Congenital Heart Disease. *J Pediatr Nurs*. 2015;30(5):e3-10.
21. Burstrom A, Ojmyr-Joelsson M, Bratt EL, et al. Adolescents With Congenital Heart Disease and Their Parents: Needs Before Transfer to Adult Care. *J Cardiovasc Nurs*. 2016;31(5):399-404.
22. Burström Å, Bratt EL, Frenckner B, et al. Adolescents with congenital heart disease: their opinions about the preparation for transfer to adult care. *Eur J Pediatr*. 2017;176(7):881-89.
23. Bratt EL, Burström Å, Hanseus K, et al. Do not forget the parents—Parents' concerns during transition to adult care for adolescents with congenital heart disease. *Child Care Health Dev*. 2017;44(2):278-84.
24. Bartholomew LK, Markham CM, Ruiter R, et al. Planning health promotion programs: an intervention mapping approach. 4th ed. San Francisco: Jossey-Bass; 2016.
25. Dolk H, Loane M, Garne E, et al. Congenital Heart Defects in EuropeClinical Perspective. *Circulation*. 2011;123(8):841-9.
26. Acuna Mora M, Sparud-Lundin C, Bratt EL, et al. Person-centred transition programme to empower adolescents with congenital heart disease in the transition to adulthood: a study protocol for a hybrid randomised controlled trial (STEPSTONES project). *BMJ Open*. 2017;7(4):e014593.
27. Acuna Mora M, Luyckx K, Sparud-Lundin C, et al. Patient empowerment in young persons with chronic conditions: Psychometric properties of the Gothenburg Young Persons Empowerment Scale (GYPES). *PloS One*. 2018;13(7):e0201007.
28. Small N, Bower P, Chew-Graham CA, et al. Patient empowerment in long-term conditions: development and preliminary testing of a new measure. *BMC Health Serv Res*. 2013;13(1):263.
29. West JF. Public health program planning logic model for community engaged Type 2 diabetes management and prevention. *Eval Program Plann*. 2014;42:43-49.
30. Carroll C, Patterson M, Wood S, et al. A conceptual framework for implementation fidelity. *Implementat Sci*. 2007;2:40.
31. Dobson D, Cook TJ. Avoiding type III error in program evaluation: Results from a field experiment. *Eval Program Plann*. 1980;3(4):269-276.
32. Jansen YJ, Foets MM, de Bont AA. The contribution of qualitative research to the development of tailor-made community-based interventions in primary care: a review. *Eur J Public Health*. 2010 Apr;20(2):220-6.
33. Creswell JW, Plano Clark VL. Designing and conducting mixed methods research. 2nd ed. Los Angeles: SAGE Publications; 2011.
34. Polit, D., & Beck, C. Nursing research : Generating and assessing evidence for nursing practice. 10th ed. Wolters Kluwer: Philadelphia; 2016.

35. Wilson AD, Onwuegbuzie AJ, Manning LP. Using paired depth interviews to collect qualitative data. *Qual Rep*. 2016;21(9):1549-73.

For peer review only - http://bmjopen.bmj.com/site/about/guidelines.xhtml

36. Griffin TL, Clarke JL, Lancashire ER, et al. Process evaluation results of a cluster randomised controlled childhood obesity prevention trial: The WAVES study. *BMC Public Health*. 2017;17(1):681.
37. Roper J, Shapira J. Ethnography in Nursing Research. London: SAGE Publications Ltd; 2000.
38. Kitzinger J. Qualitative research. Introducing focus groups. *BMJ*. 1995;311(7000):299-302.
39. Krippendorff K. Content analysis: An introduction to its methodology. Sage; 2012.40. Tong A, Sainsbury P, Craig J. Consolidated criteria for reporting qualitative research (COREQ): a 32-item checklist for interviews and focus groups. *Int J Qual Health Care*. 2007;19(6):349-57.
41. Graneheim UH, Lundman B. Qualitative content analysis in nursing research: concepts, procedures and measures to achieve trustworthiness. *Nurse Educ Today*. 2004;24(2):105-12.
42. O'Cathain A, Murphy E, Nicholl J. The quality of mixed methods studies in health services research. *J Health Serv Res Policy*. 2008;13(2):92-8.
43. O'Cathain A, Murphy E, Nicholl J. Three techniques for integrating data in mixed methods studies. *BMJ*. 2010;341:c4587.
44. World Medical Association Declaration of Helsinki: ethical principles for medical research involving human subjects. *JAMA*. 2013;310(20):2191-4.
45. Eldh AC, Olai L, Jönsson B, et al. Supporting first-line managers in implementing oral care guidelines in nursing homes. *Nord J Nurs Res*. 2017:2057158517713379.
46. Lloyd J, Dean S, Creanor S, et al. Intervention fidelity in the definitive cluster randomised controlled trial of the Healthy Lifestyles Programme (HeLP) trial: Findings from the process evaluation. *Int J Behav Nutr Phys Act*. 2017;14(1):163.
47. Dusenbury L, Brannigan R, Falco M, et al. A review of research on fidelity of implementation: implications for drug abuse prevention in school settings. *Health Educ Res*. 2003;18(2):237-56.
48. Walton H, Spector A, Tombor I, et al. Measures of fidelity of delivery of, and engagement with, complex, face-to-face health behaviour change interventions: A systematic review of measure quality. *Br J Health Psychol*. 2017;22(4):872-903.
49. Morgan-Trimmer S, Wood F. Ethnographic methods for process evaluations of complex health behaviour interventions. *Trials*. 2016;17(1):232.
50. Steckler AB, Linnan L, Israel B. Process evaluation for public health interventions and research: Jossey-Bass: San Francisco; 2002.
51. Landsberger HA. Hawthorne Revisited: Management and the Worker, Its Critics, and Developments in Human Relations in Industry. 1958.

For peer review only - http://bmjopen.bmj.com/site/about/guidelines.xhtml

Figure legends

TC: Transition coordinator

Figure 1: Key components of STEPSTONES transition program and implementation steps

RCT: randomized controlled trial; TC: transition coordinator

Figure 2: Overview of the STEPSTONES RCT-study design

Figure 3: Overview of the process evaluation of the STEPSTONES transition program, adapted from the Medical Research Council's guidance

Figure 4: Overview of STEPSTONES process evaluation components and data sources

For peer review only

For peer review only - http://bmjopen.bmj.com/site/about/guidelines.xhtml

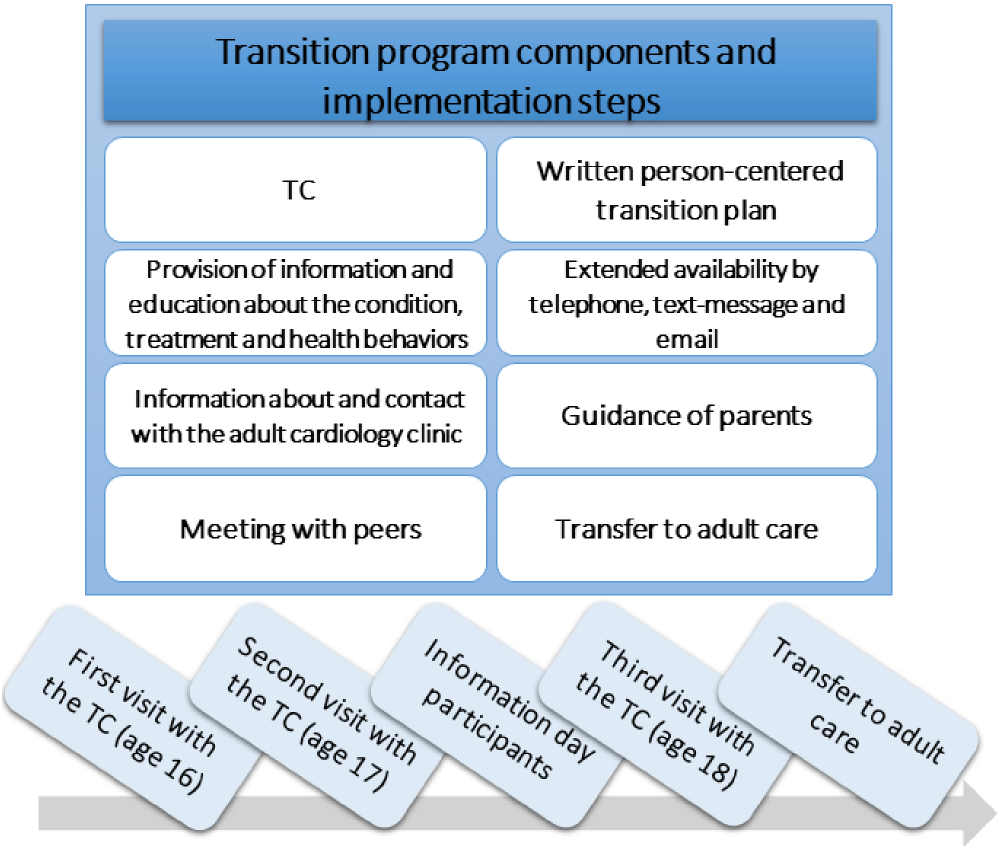

TC: Transition coordinator
Figure 1: Key components of STEPSTONES transition program and implementation steps

58x50mm (300 x 300 DPI)

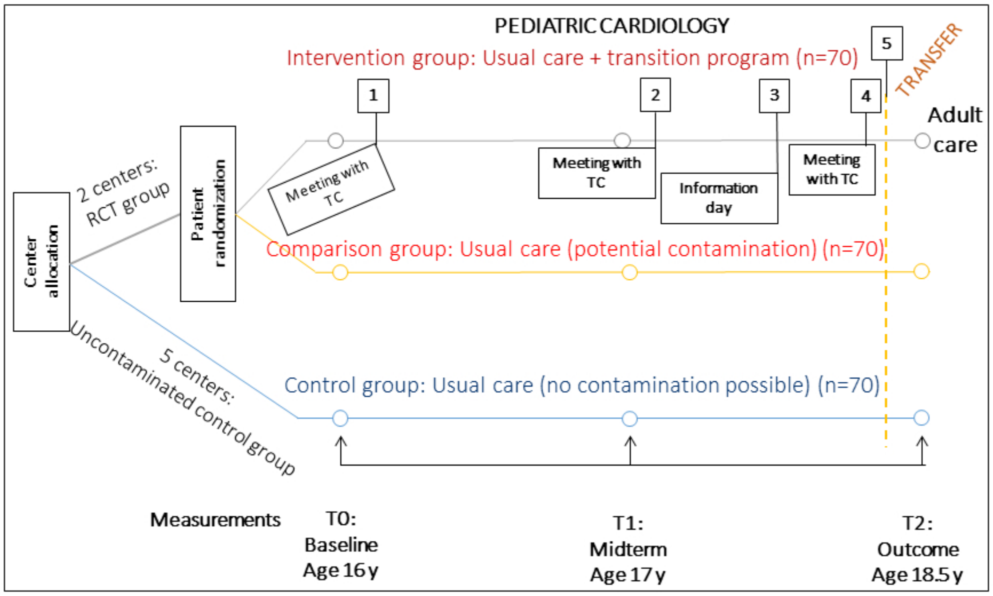

RCT: randomized controlled trial
Figure 2: Overview of the STEPSTONES RCT-study design

73x44mm (300 x 300 DPI)

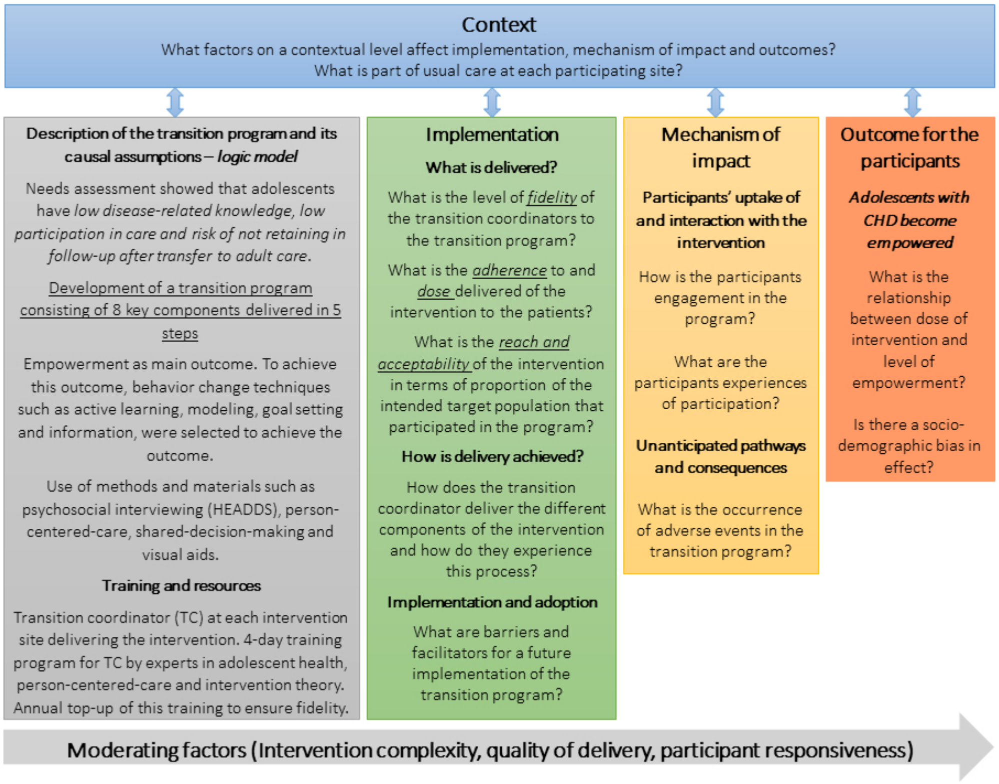

Figure 3: Overview of the process evaluation of the STEPSTONES transition program, adapted from the Medical Research Council's guidance

76x60mm (300 x 300 DPI)

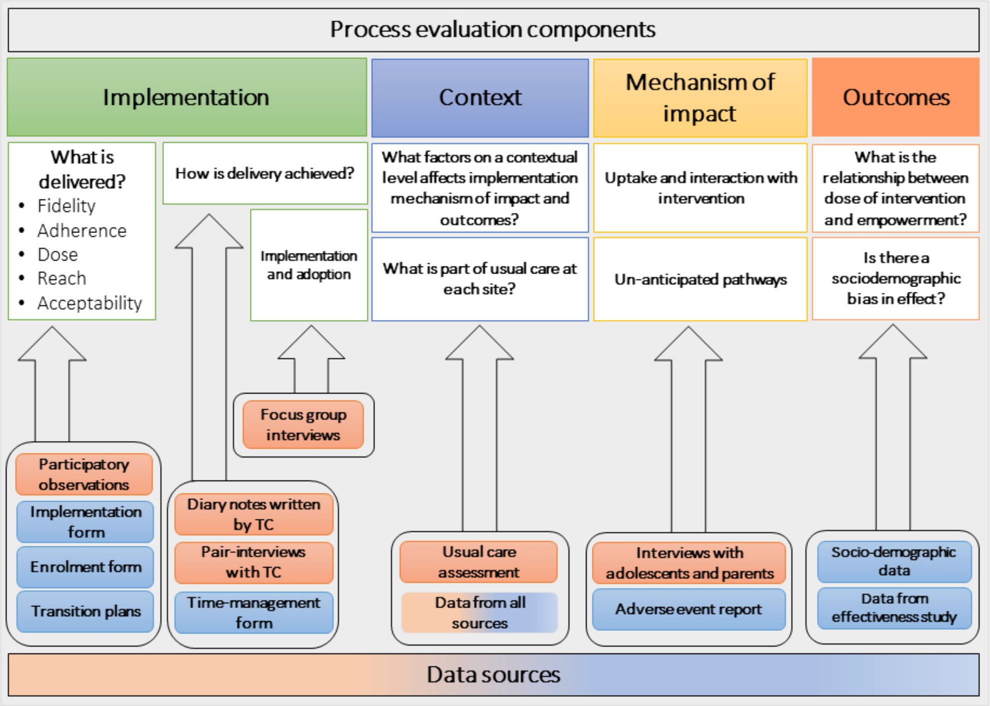

Figure 4: Overview of STEPSTONES process evaluation components and data sources

78x55mm (300 x 300 DPI)
